# Compositional flexibility in Li–N–H materials: implications for ammonia catalysis and hydrogen storage†

**DOI:** 10.1039/d1cp02440j

**Published:** 2021-07-05

**Authors:** Joshua W. Makepeace, Jake M. Brittain, Alisha Sukhwani Manghnani, Claire A. Murray, Thomas J. Wood, William I. F. David

**Affiliations:** School of Chemistry, University of Birmingham Edgbaston B15 2TT UK j.w.makepeace@bham.ac.uk; Inorganic Chemistry Laboratory, Department of Chemistry, University of Oxford South Parks Road Oxford OX1 3QR UK; ISIS Pulsed Neutron and Muon Facility, Rutherford Appleton Laboratory Harwell Campus OX11 0QX UK; Diamond Light Source Harwell Campus OX11 0DE UK

## Abstract

Li–N–H materials, particularly lithium amide and lithium imide, have been explored for use in a variety of energy storage applications in recent years. Compositional variation within the parent lithium imide, anti-fluorite crystal structure has been related to both its facile storage of hydrogen and impressive catalytic performance for the decomposition of ammonia. Here, we explore the controlled solid-state synthesis of Li–N–H solid-solution anti-fluorite structures ranging from amide-dominated (Li_4/3_(NH_2_)_2/3_(NH)_1/3_ or Li_1.333_NH_1.667_) through lithium imide to majority incorporation of lithium nitride–hydride (Li_3.167_(NH)_0.416_N_0.584_H_0.584_ or Li_3.167_NH). Formation of these solid solutions is demonstrated to cause significant changes to the thermal stability and ammonia reactivity of the samples, highlighting the potential use of compositional variation to control the properties of the material in gas storage and catalytic applications.

## Abstract 2

A wide solid solution based on the lithium imide anti-fluorite structure is demonstrated and related to its energy storage functions.

## Graphical abstract

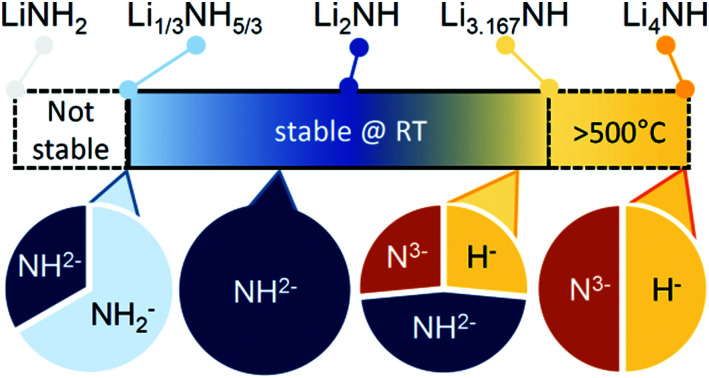

## Introduction

Compositional variation, and the ability to exert synthetic control over such variation, lies at the heart of much of functional materials chemistry research, impacting a wide range of materials properties from metal alloy strength to superconductivity. The ability of solid structures to accommodate variable quantities of metal ions is central to lithium-ion battery chemistry, which has transformed the field of portable devices and is currently changing the landscape of passenger transportation.

Variable anion content is at the heart of applications of metal–nitrogen–hydrogen solid state materials. The Li–N–H materials system consists of lithium salts of anions formed from nitrogen and/or hydrogen, and is most well-known for its favourable hydrogen storage properties.^1,2^ The hydrogenation of lithium nitride (Li_3_N) produces lithium imide (Li_2_NH) and then lithium amide (LiNH_2_), with stoichiometric equivalents of lithium hydride (LiH) (eqn (1)). These reactions gained attention in the context of lightweight hydrogen stores for transport applications^3–5^ because of the relative ease with which reversible amide–imide conversion could be achieved in comparison with other complex anion based hydrogen stores.^6,7^

1 Li_3_N + 2H_2_ → Li_2_NH + LiH + H_2_ → LiNH_2_ + 2LiH

Mixed amide–imide phases (Li_2−*x*_(NH_2_)_*x*_(NH)_1−*x*_ or Li_2−*x*_NH_1+*x*_, 0 < *x* < 1) were observed during hydrogen storage and release reactions, and facilitate a smooth structural transformation between hydrogenated and dehydrogenated states.^8,9^ By analogy with interstitial metal hydrides, such as PdH_0.8_, this provides an explanation for the facile hydrogen storage observed in the system.

More recently, lithium imide has also sparked interest as a highly active catalyst for the decomposition of ammonia to produce hydrogen (and nitrogen). Ammonia decomposition is likely to become increasingly important as a component of renewable energy export markets, where hydrogen produced by water electrolysis is converted to ammonia and shipped to areas with energy demand.^10,11^ Partial or complete conversion of ammonia to hydrogen allows for flexible end-use of the stored energy in combustion engines, gas turbines and fuel cells.^12–14^ Lithium imide based catalysts have shown comparable or superior performance to state-of-the-art ruthenium catalysts.^15–18^ In this context, *in situ* neutron powder diffraction experiments indicated that the lithium imide becomes non-stoichiometric under operating conditions, with a mixed amide–imide composition dependent on the temperature and conversion.^15^ Isotope exchange experiments have confirmed that this variable composition allows for bulk nitrogen and hydrogen exchange between the catalyst and ammonia.^19^

The broader significance of compositional variation in M–N–H materials is illustrated by recent reports of their use as strong promoters for ammonia synthesis in ruthenium catalysts.^20–22^ The formation of solid solutions of electride material dicalcium nitride (Ca_2_N) and calcium nitride hydride (Ca_2_NH) under ammonia synthesis conditions has been hypothesised to promote high activity through (i) the formation of a defect structure which lowers the material work function, increasing electron donation to the ruthenium particles and (ii) the ability to act as a reservoir for hydrogen, avoiding the inhibition of catalytic activity by hydrogen which is often observed for ruthenium catalysts.^22^

**Fig. 1:**
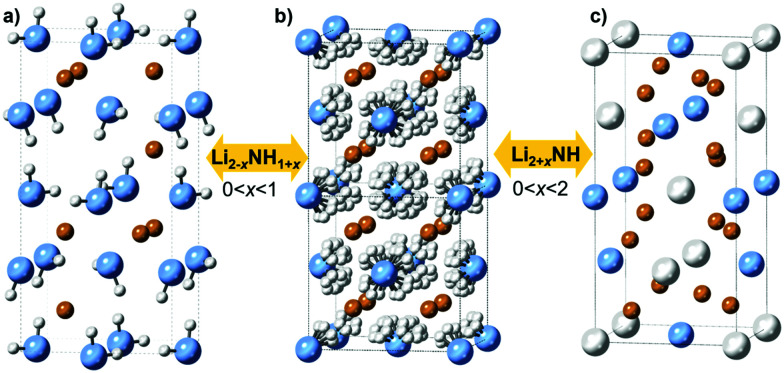
Crystal structures of (a) lithium amide (b) lithium imide with disordered hydrogen positions (>80 °C) and (c) lithium nitride–hydride, with solid solutions indicated in between structures. Lithium is shown in orange, nitrogen in blue, and hydrogen in white.

It is clear that the impressive performance of the M–N–H materials in these applications relates to compositional flexibility, which derives from the close structural relationships between the phases involved. Lithium amide and lithium imide both have crystal structures based on a cubic close packed arrangement of anions with occupancy of the tetrahedral holes by cations: the anti-fluorite structure. Lithium amide has a layered arrangement of lithium ions occupying half the holes, while in lithium imide these holes are fully occupied (Fig. 1a and b).

Structural similarities are also clear between lithium imide and lithium nitride–hydride (Li_4_NH, Fig. 1c). Here, nitride and hydride ions form an ordered arrangement based on the cubic close packed lattice, resulting in an *a* × *a* × 2*a* supercell of the anti-fluorite structure; the charge imbalance between nitride (3−) and hydride (1−) displaces the lithium ions from the tetrahedral site towards a trigonal coordination. As such, lithium nitride–hydride could also be described as an anti-scheelite structure, which is an ordered variant of the anti-fluorite structure. This close structural relationship between anti-fluorite and anti-scheelite suggests that mixed lithium imide–nitride–hydride phases (Li_2+*x*_(NH)_1−*x*_N_*x*_H_*x*_ or Li_2+*x*_NH, 0 < *x* < 2) can be formed. This possibility was first reported by Marx, but without accompanying experimental data.^23^ However, evidence for the existence of such phases has been published in the context of hydrogen storage, where a cubic phase termed “quasi imide” was reported in neutron powder diffraction studies of the low-pressure hydrogenation of lithium nitride.^24–26^ The presence of imide–nitride–hydride was also proposed as an explanation for the ammonia decomposition activity of lithium imide, given the formation and subsequent ammoniation of lithium nitride–hydride within the imide structure is a possible ammonia decomposition cycle:^19^

2 2Li_2_NH → Li_4_NH + 1/2H_2_ + 1/2N_2_

3 Li_4_NH + NH_3_ → 2Li_2_NH + H_2_

Given the importance of non-stoichiometric M–N–H materials to a range of applications, there are very few studies which seek to synthesise and study these solid solutions. In this work, we explore routes to the synthetic control of the stoichiometry in the Li–N–H system and the variation of properties which result. We aim to provide a synthetically-accessible library of Li–N–H materials which can be further investigated for their functional properties as catalysts, catalyst supports and hydrogen stores, as well as synthetic approaches for the synthesis of other functional M–N–H solid solutions.

## Experimental

### Sample handling

All sample handling was performed in an argon-filled glove box (MBraun Unilab, O_2_ < 0.1 ppm, H_2_O < 0.1 ppm) because of the water sensitivity of the samples.

### Synthesis of lithium amide–imide (Li_2−*x*_NH_1+*x*_) samples

13 samples of Li_2−*x*_NH_1+*x*_ (where *x* = 0, 0.083, 0.167, 0.250, 0.333, 0.417, 0.500, 0.583, 0.667, 0.750, 0.833, 0.917, 1.000) were synthesised by the reaction of lithium amide (LiNH_2_, Sigma Aldrich, 95%) with lithium nitride (Li_3_N, Sigma Aldrich, >99.5%) or lithium hydride (LiH, Sigma Aldrich, 95%) according to the following reactions:

4 (1 − *x*)Li_3_N + (1 + *x*)LiNH_2_ → 2Li_2−*x*_NH_1+*x*_

5 (1 − *x*)LiH + LiNH_2_ → Li_2−*x*_NH_1+*x*_ + (1 − *x*)H_2_

The nitride-based method was the most reliable in producing samples of the desired stoichiometry; the hydride reaction was found to be more likely to show incomplete reaction, as evidenced by lack of formation of the low-temperature (*Fd*3̄*m*) lithium imide phase for the *x* = 0 sample (Fig. S1, ESI†). While the data discussed in the main text are from the nitride samples, very similar results were observed for the hydride samples (Fig. S1–S4, ESI†).

The reagents were weighed out according to the desired ratio, with a total sample mass of 1 g. The samples were hand-ground using an agate mortar and pestle for approximately five minutes to ensure adequate mixing, and then transferred into a tungsten carbide milling jar (volume = 125 mL). Tungsten carbide milling balls (3 mm diameter) were added to achieve a 40 : 1 mass ratio with the powder. The jar was then sealed and transferred into a planetary ball mill (Retsch PM100) and milled for 1 hour at 150 rpm, with the direction of rotation reversed at 10 minutes intervals.

0.5 g of the resultant mixture was transferred into a cylindrical alumina crucible and placed in the bottom of a glass boiling tube fitted with a Young's tap T-piece to prevent air ingress. The sample was then heated in a tube furnace (Lenton LTF12/38/500) under a continuous flow of argon to 300 °C at a heating rate of 2 °C min^−1^, and then dwelled at that temperature for 12 hours.

### Synthesis of lithium imide–nitride–hydride (Li_2+*x*_NH) samples

13 samples of Li_2+*x*_NH (where *x* = 0, 0.167, 0.333, 0.500, 0.667, 0.833, 1.000, 1.167, 1.333, 1.500, 1.667, 1.833, 2.000) were synthesised by the solid state reaction of lithium imide with lithium nitride–hydride according to the following reaction:

6 (2 − *x*)Li_2_NH + *x*Li_4_NH → 2Li_2+*x*_NH

Lithium imide for the reaction was synthesised as for the Li_1+*x*_NH_2−*x*_ samples (*x* = 1.000). Lithium nitride–hydride was synthesised by the reaction of lithium hydride (Sigma Aldrich, 95%) and lithium nitride (Sigma Aldrich, >99.5%):

7 LiH + Li_3_N → Li_4_NH

The reactants were hand-ground and then milled according to the procedure outlined above. 0.5 g of the resultant mixture was pelletised using a hand press (Pike Technologies, 7 mm diameter), and placed into a nickel crucible in a quartz boiling tube, sealed with a Young's tap T-piece. The sample was heated to 500 °C at 2 °C min^−1^ and dwelled for 12 hours.

Synthesis of the mixed Li_2+*x*_NH samples from lithium imide and lithium nitride–hydride was the same as for the other samples, except that the milling procedure used a rotation rate of 400 rpm, and the furnace temperature set point was 540 °C.

### X-Ray diffraction

Structural analysis of the sample was performed using synchrotron X-ray diffraction; data were collected on the I11 high-resolution powder diffraction beamline (*λ* = 0.826021 Å) at the Diamond Light Source.^27^ Powder samples were loaded into 0.7 mm borosilicate glass capillaries, which were flame sealed. The samples were mounted onto the diffractometer using a robotic arm, and rotated while diffraction data (8–20 minutes scans) were collected using the five Multi-Analyser Crystal (MAC) detectors.

All XRD data were analysed (generally by Rietveld analysis) using the TOPAS Academic package (v6).^28^

### Raman spectroscopy

Spectroscopic analysis of the samples was performed using a Raman microscope (Bruker Senterra). Each sample was loaded into a 0.7 mm wide borosilicate glass capillary to a height of approximately 10 mm and sealed with grease. Each capillary was then individually loaded onto the adjustable table, moved into position and brought into focus using the built-in microscope with ×10 lens. Raman data were collected using a 20 mW, 532 nm laser, with an aperture of 50 × 1000 μm to ensure sampling across multiple powder grains. Peak areas and positions were analysed using the TOPAS Academic package.

### Thermal analysis

The thermal behaviour of the samples was analysed by differential scanning calorimetry (DSC), with experiments run on a Mettler Toledo DSC 1 instrument. 10 mg of powdered sample was loaded into a 20 μL aluminium pan and sealed with an aluminium lid in the glove box. After loading onto a carousel, a robotic arm individually loaded each sample into an inert atmosphere, pierced the lid and started the heating cycle. For amide–imide samples, each sample was heated to 450 °C from 25 °C at a rate of 5 °C min^−1^, then immediately cooled back to 25 °C at a rate of 10 °C min^−1^. Each imide–nitride–hydride sample was heated to 575 °C at a rate of 10 °C min^−1^, dwelled for 10 minutes and cooled back down to 100 °C. A flowing argon atmosphere (50 cm^3^ min^−1^) was used throughout each experiment.

The reactivity of the lithium imide–nitride–hydride samples when heated under ammonia was studied in a simultaneous thermal analysis (STA) apparatus (Netzsch F1 Jupiter). Each sample was loaded into a 20 μL aluminium pan, sealed with an aluminium lid and pierced before loading to allow gas exchange. The samples were heated to 350 °C under ammonia at a rate of 5 °C min^−1^, dwelled at temperature for 5 hours and cooled back down to room temperature. The instrument was then purged with inert gas to remove the sample safely.

## Results and discussion

### Structural analysis: average structure

**Fig. 2:**
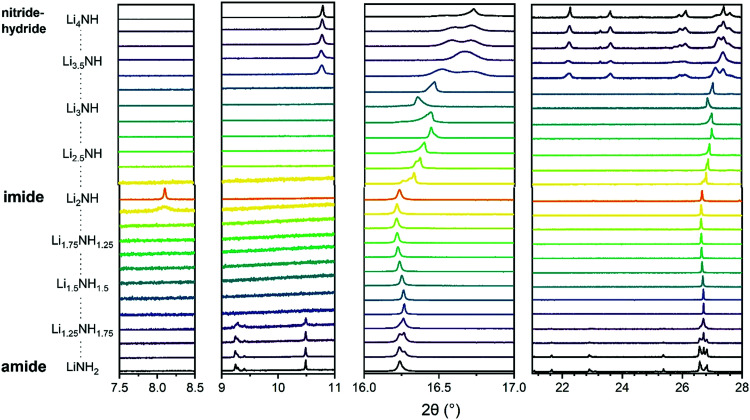
Sections of synchrotron powder X-ray diffraction data for the Li–N–H samples. The first two panels show the superlattice regions where peaks for tetragonal lithium amide (9–10.5°) or lithium nitride–hydride (11°) and *Fd*3̄*m* lithium imide (8°) are expected. The third panel shows the peak region for (111)/(112) reflections of the cubic/tetragonal phases. The fourth panel shows a broader region highlighting the tetragonal peaks present for lithium amide and lithium nitride–hydride. Full diffraction patterns with Rietveld analysis are available in Fig. S5–S29 (ESI†).

Synchrotron powder X-ray diffraction data provides a basis for assessment of the structural variation across the Li–N–H samples and the successful formation of solid solutions. These data are shown in Fig. 2. Lithium imide, shown in the central pattern in Fig. 2, is observed to form the low-temperature *Fd*3̄*m* structure which is a 2 × 2 × 2 supercell of the antifluorite structure, formed as a result of ordered N–H bond orientations facilitated by the formation of a Frenkel defect by 1/8 of the lithium ions. This structure is most obviously evidenced by presence of the (111) peak at around 8°. It can be seen that this ordered structure is specific to samples very close to Li_2_NH stoichiometry; the peak is not observed for any of the imide–nitride–hydride samples, and in the Li_1.917_NH_1.083_ amide–imide sample, it is present as a weak and broad feature, indicative of a coherence length of around 300 Å.

**Fig. 3:**
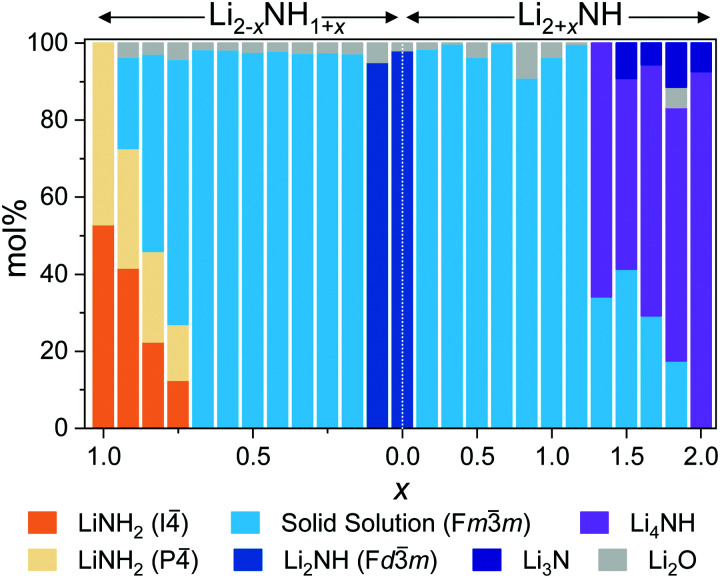
Phase composition as determined by Rietveld analysis of synchrotron powder X-ray diffraction data.

On either side of the stoichiometric imide, there is a wide compositional range where the diffraction data show only *Fm*3̄*m* peaks, indicating a disordered (solid solution) average structure. The amide–imide samples show a single well-defined phase up to Li_1.333_NH_1.667_ (*x* = 0.667), two-thirds substitution of amide within the structure. A similar trend was observed for amide–imide samples synthesised *via* the lithium amide lithium hydride reaction. This reflects an improved synthesis protocol over previous attempts to synthesise amide–imide phases, where a single phase was only observed^29^ up to *x* = 0.4. For imide–nitride–hydride samples, an *Fm*3̄*m* structure is observed up to Li_3.167_NH (*x* = 1.167). These samples displayed much more complex microstructure than the amide–imide samples, with asymmetric peak shapes that are likely to be indicative of regions of variable composition. This likely reflects the higher enthalpic barriers to ion migration in the nitride–hydride–imide array compared with the amide–imide, where a lower-energy proton exchange and lithium ion migration is required. Grinding and re-heating these samples did not improve the peak symmetry. While the use of higher temperatures to synthesise those samples risks thermal degradation (and therefore loss of compositional control), further optimisation of milling routines may yet yield more uniform solid solutions. The data in the imide–nitride–hydride samples were modelled with a series of solid solution *Fm*3̄*m* phases with a constrained lattice parameter – composition relationship in the Rietveld analysis (ESI,† Section S3), while only one such phase was required to fit the amide–imide samples. The resultant phase compositions are shown in Fig. 3.

Moving outside of the region where a full solid solution is formed, tetragonal lithium amide and lithium nitride–hydride phases are observed, with diminishing proportions of the cubic solid solution phases. In the amide-rich samples, additional diffraction peaks were observed in the superlattice region: peaks at 9.28° and 9.4° in addition to the (002) peak of *I*4̄ lithium amide at 9.24°. These peaks were indexed to a *P*4̄ structure (details in Table S1, ESI†) with very similar lattice parameters to lithium amide; consequently the data were modelled with a mixture of *I*4̄ and *P*4̄ lithium amide.

The loss of body-centring implied by the *P*4̄ phase is most likely related to a change in the lithium occupancy in the layered structure. The *I*4̄ structure has an alternating 0.25/0.75 occupancy of the tetrahedral sites in layers along the *c*-axis. Refinement of the crystallographically unique lithium sites in the *P*4̄ structure indicates a disruption of that ordering (Table 1). The precise nature of the disordering is unclear, as the proportion of the *P*4̄ phase is not sensitive to the overall sample stoichiometry, and the refined stoichiometry (Li_0.96(6)_NH_2.04(6)_) is not significantly different from lithium amide. It is likely that this phase represents a kinetically stabilised phase, perhaps as a result of the synthesis procedure; further examination of its thermal stability may confirm this.

**Table 1:** Fractional occupancy of tetrahedral holes by lithium along the (001) direction in *I*4̄ and *P*4̄ lithium amide. The resultant average stoichiometry of the phase is also shown

Li layer fractional occupancy		
*c* axis position	*I*4̄	*P*4̄
0.75	0.250	0.32(7)
0.50	0.750	0.6(1)
0.25	0.250	0.32(7)
0.00	0.750	0.7(1)
Stoichiometry	LiNH_2_	Li_0.96(7)_NH_2.04(7)_

**Fig. 4:**
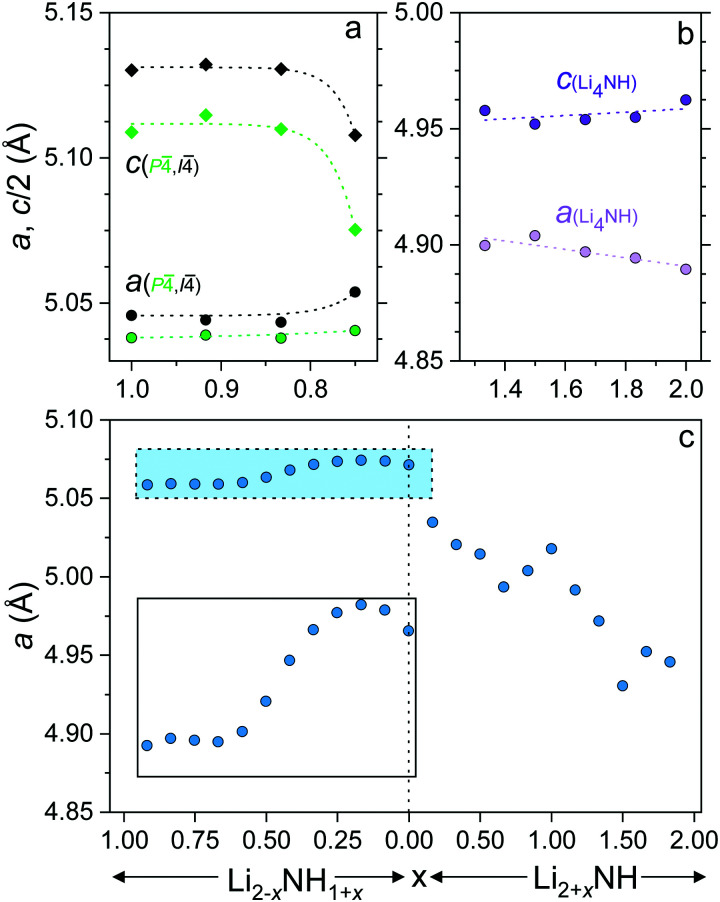
Lattice parameters of (a) lithium amide (b) lithium nitride–hydride and (c) *Fm*3̄*m* phases (and 0.5 × *a* for the *Fd*3̄*m* Li_2_NH phase), with an inset plot of the highlighted amide–imide region. Error bars are smaller than the data points.

In order to gain insight into the nature of the compositional variation across the samples, lattice parameter data for the different phases are shown in Fig. 4. The lattice parameter value reported for the *Fm*3̄*m* phase in the imide–nitride–hydride samples is a weighted average of the three lattice parameters of the fitted phases.

The lithium amide lattice parameters (Fig. 4a) are stable for 0.833 ≤ *x* ≤ 1, but show a drop in *c*/*a* for the *x* = 0.750 sample, which may reflect some compositional variation within the tetragonal structures. Conversely, the lithium nitride–hydride structure shows a steady drop in *c*/*a* as the composition moves away from stoichiometric Li_4_NH, which is likely to be associated with partial site disordering with imide incorporation. Indeed, initial reports of the structure of lithium nitride–hydride *via* powder diffraction measurements refined ∼5% anion site disorder,^23^ while single crystal diffraction data indicated a perfectly ordered structure.^30^

The lattice parameter of the *Fm*3̄*m* phase in the amide–imide samples displays an apparent minimum at *x* = 0.667 (Li_1.333_NH_1.667_), consistent with a maximum level of amide incorporation in the solid solution phase; further amide remains in the tetragonal structure. Between *x* = 0.583 and *x* = 0.333 the lattice parameter change is approximately linear before tailing off to a maximum at *x* = 0.167 and decreasing for Li_1.917_NH_1.083_ and Li_2_NH. This would appear to reflect the imide proportion at which onset of the ordered imide structure occurs, which relies on the ability to form lithium vacancies that are tetrahedrally coordinated by four imide groups.^31,32^

The lattice parameter trend for the imide–nitride–hydride samples is more complex. A small proportion of nitride–hydride incorporation produces a more substantial response in the solid solution lattice parameter given the larger lattice mismatch for Li_2_NH/Li_4_NH than Li_2_NH/LiNH_2_. The lattice parameter of Li_3_NH appears to break the monotonous trend in the lattice parameter; this may reflect local ordering of the anions given the equal quantities of NH^2−^ and (N^3−^ + H^−^) at this composition, though no evidence of long-range ordering was observed. Additionally, the lattice parameter does not simply level off after *x* = 1.167, whereupon further increasing the proportion of nitride–hydride results in tetragonal lithium nitride–hydride formation. Instead, it continues to decrease, implying a continuing change in composition, though possibly at a less rapid rate, which would imply a reduced nitride–hydride component compared to the overall stoichiometry of the sample. It is also clear from an examination of the diffraction data that the microstructure of the solid solution phase and lithium nitride–hydride in these samples differs from the other imide–nitride–hydride mixtures. The Bragg peaks of both phases are significantly strain-broadened. One possible interpretation of these data is that a solid solution is possible across the whole compositional range, but that in these samples, the solid solution is only stable at elevated temperatures, and that rapid separation upon cooling leads to two highly strained, inhomogeneous phases. This possibility will be discussed further in relation to the thermal analysis of the samples.

### Spectroscopic analysis

**Fig. 5:**
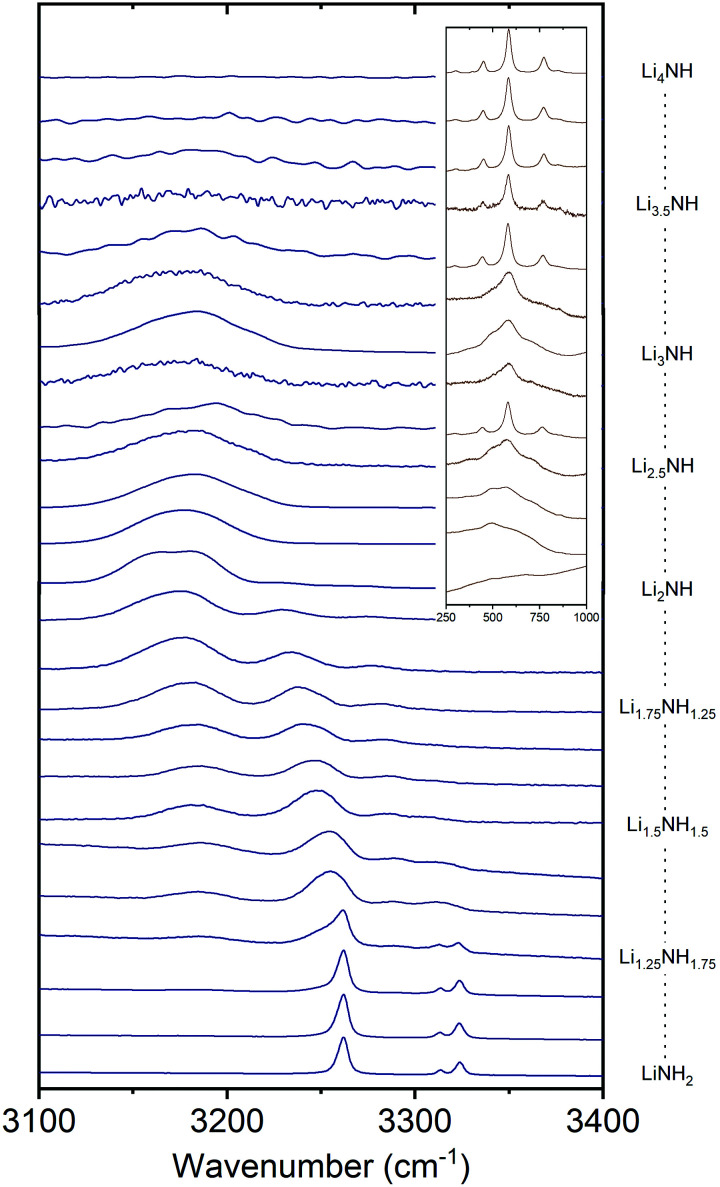
The N–H stretching region of the Raman spectra of the Li–N–H samples, and inset, the low-energy region of the imide–nitride–hydride samples.

Raman spectroscopy was used in order to provide an insight into the local environments of the NH_*x*_ groups in the samples, with the spectra shown in Fig. 5. The key features of the stoichiometric compounds are:

LiNH_2_ – symmetric NH_2_ stretch at 3262 cm^−1^, asymmetric NH_2_ stretches at 3314 and 3324 cm^−1^.

Li_2_NH – broad NH stretch at 3180 cm^−1^.

Li_4_NH – sharp phonon modes at 452, 584 and 775 cm^−1^.

Analysis of the Raman spectra of the amide–imide samples is based on a similar approach to that previously reported:^29^ the tetragonal lithium amide gives sharp NH_2_ stretch signals which remain at constant wavenumber, reflecting the compositional stability of the phase. A second set of broadened amide stretch peaks can be observed which represent amide ions in solid solution; these are the only amide peaks present from Li_1.333_NH_1.667_ onwards, reflecting the observations from the XRD data. These peaks, and the imide peak, can be observed to move to lower wavenumber as the solid solution becomes more imide-like and the average coordination number of lithium ions around each NH_*x*_ group increases (peak areas and positions are given in the Fig. S31 and S32, ESI†). These data indicate the formation of a well-mixed solid solution.

In the case of imide–nitride–hydride samples, this trend is much less clear, given that the lithium occupancy does not vary across these samples. On the whole, the width of the imide stretch peak is larger when compared with the amide–imide samples, which likely reflects a wider compositional range in these samples, which is consistent with the peak shapes in the X-ray diffraction data. The lithium nitride–hydride peaks are well-defined in the samples which contain tetragonal lithium nitride, and generally much broader in the solid–solution samples.

### Thermal analysis

**Fig. 6:**
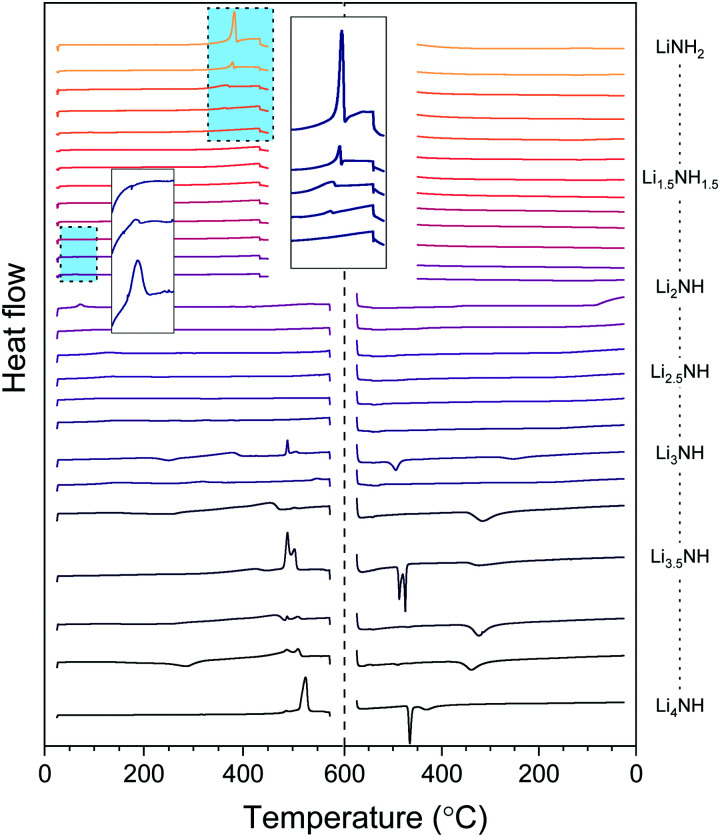
Differential scanning calorimetry data for the Li–N–H samples. An endothermic event is a positive change in the heat flow. Inset are the highlighted regions for the melting of lithium amide and the phase transition of lithium imide.

The thermal stability of the solid solutions is critical to determining the applications to which they might be best suited. In order to assess this, the samples were analysed by differential scanning calorimetry, with the data showing both heating and cooling cycles in Fig. 6. The amide–imide samples were tested between 25 °C and 450 °C, given the known decomposition of lithium amide to lithium imide around 400 °C, while the imide–nitride–hydride samples were tested up to 575 °C, given the decomposition of lithium imide to lithium nitride hydride above 600 °C.

The melting of lithium amide is seen as a sharp endotherm at 370 °C, just prior to the onset of decomposition to lithium imide (shown in the larger inset box in Fig. 6). This melt transition is rapidly suppressed upon introduction of lithium imide, and is only visible at all for the samples which contain a detectable proportion of tetragonal lithium amide (LiNH_2_ to Li_1.333_NH_1.667_). All the remaining amide–imide solid solutions display no significant thermal events on heating or cooling, aside from the *Fd*3̄*m* to *Fm*3̄*m* phase transition in Li_1.913_NH_1.083_ and Li_2_NH at 60 °C, highlighted in the small inset box in Fig. 6. This trend continues for the imide–nitride–hydride samples for which only *Fm*3̄*m* phases were observed (Li_2_NH to Li_3.167_NH aside from Li_3_NH).

For the samples which had both solid solution and tetragonal Li_4_NH phases present in the diffraction data, the thermal behavior is more complex. Endothermic events at 489 °C can be seen in these samples, followed by a second endotherm from 505–510 °C. A tetragonal to cubic phase transition for Li_4_NH has previously been reported at 497 °C,^33^ and so we hypothesise that these two endotherms may be this phase transition followed by formation of a single imide–nitride–hydride phase. Interestingly, the endotherm for Li_4_NH is at significantly higher temperature (525 °C), though has a large hysteresis, with the corresponding exotherm on cooling observed at 465 °C. The Li_3_NH and Li_3.5_NH samples also display exotherms in this region, perhaps indicating incomplete mixing of imide and nitride–hydride. For the other samples in this compositional range, a broad exotherm is observed in the 315–340 °C range. It is probable that this feature corresponds to phase segregation of the high temperature solid solution into the *Fm*3̄*m* and tetragonal Li_4_NH phases observed in the diffraction data. This is consistent with the observed broadened Bragg peaks for these phases as discussed earlier.

### Implications for hydrogen storage and catalysis

**Fig. 7:**
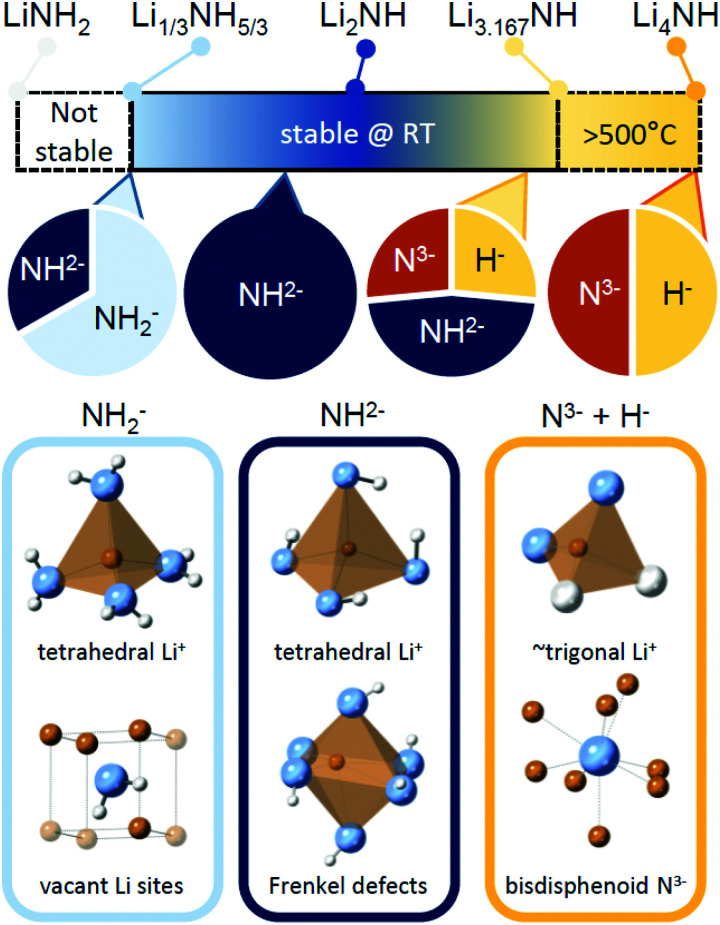
A summary of the stable composition range of the Li–N–H solid solution, along with key structural features expected for samples dominate by amide, imide or nitride–hydride anions.

The Li–N–H solid solution demonstrates remarkable tolerance for compositional variation, with samples successfully synthesised for the composition range Li_1.333_NH_1.667_ to Li_3.167_NH, and indications of wider stability for nitride–hydride rich samples above 500 °C. A summary of the stable composition range and some of the local structural features which characterise different regions of the solid solution are given in Fig. 7. The structural and thermal stability data collected for these samples provide some useful insights into the properties of the solid solution, which may impact use of the material in hydrogen storage and catalysis applications.

In the field of hydrogen storage, the reversibility of the hydrogen storage process has been attributed to solid solution formation. This process is facilitated by Li^+^ and H^+^ diffusion through the structure.^8,34^ However, the temperature for reversible hydrogen storage is still significantly higher than could be achieved using waste heat from a PEM fuel cell, reducing overall efficiency. Given the tetragonal amide structure is known to be a very poor Li^+^ conductor (∼10^−10^ S cm^−1^ at room temperature^35^), it might be expected that the hydrogen storage properties would be improved by keeping the system within the more conductive lithium imide structure (Li_2_NH has been reported to have a room temperature conductivity of around 3 × 10^−4^ S cm^−1 ^^35,36^). Having revealed an apparent upper limit to amide content of the solid solution at Li_1.333_NH_1.667_, it may be possible to achieve a more favourable operating temperature by restricting the hydrogenation capacity to two thirds of the theoretical maximum.

Loss of the tetragonal structure is also important to the deployment of lithium amide–imide catalysts for ammonia decomposition, where loss of catalyst material through melting will degrade activity over time and also thwart attempts to create high surface area formulations to maximise turnover frequency. In our recent demonstration of a lithium imide ammonia cracking unit coupled to a PEM fuel cell, a Dreschel bottle style reactor was used to avoid blocking of the reactor in case of catalyst melting.^37^ It would be preferable to use a packed bed reactor in order to maximise the exposure of ammonia to the catalyst surface. While the precise stoichiometry has been shown to vary under the reaction conditions chosen, it is clear that maintaining a more imide-like stoichiometry than Li_1.33_NH_1.667_ will prevent melting of the sample.

The demonstrated ability of the anti-fluorite Li–N–H structure to accommodate a wide variety of ions also has implications for understanding its role in catalysis. Certainly, the existence of a wide range of stable imide–nitride–hydride solid solutions supports the possibility that nitride–hydride formation could play a role in the catalytic decomposition of ammonia by lithium imide. Additionally, the accommodation of H^+^, H^−^, N^3−^, NH^2−^ and NH_2_^−^ within the structure suggests that these phases could play an analogous role to that ascribed to the Ca–N–H supports for ruthenium based ammonia synthesis catalysts, acting as a reservoir of reactive species which might otherwise occupy surface sites on the metal catalyst. Similarly, an analogy may be drawn to the metal nitride catalysts such as Co_3_Mo_3_N and Li–Mn–N phases, where the proposed Mars–van Krevelen mechanism involves reactivity of the lattice nitrogen in ammonia synthesis.^38–40^ The bulk transport of ions within the solid may also help explain the effectiveness of the materials as catalysts/supports despite generally low surface areas. Previous work has demonstrated that bulk nitrogen/hydrogen exchange is possible with ammonia, but these solid solutions also react with hydrogen, and lithium nitride–hydride is also able to fix nitrogen.^23,33^ Thus these phases show promise not only as catalysts in their own right, but also as components of composite catalyst formulations, where multi-site approaches are increasingly seen as the most promising way to escape the bottleneck of energy scaling relations on single-site catalyst surfaces.^41–43^

**Fig. 8:**
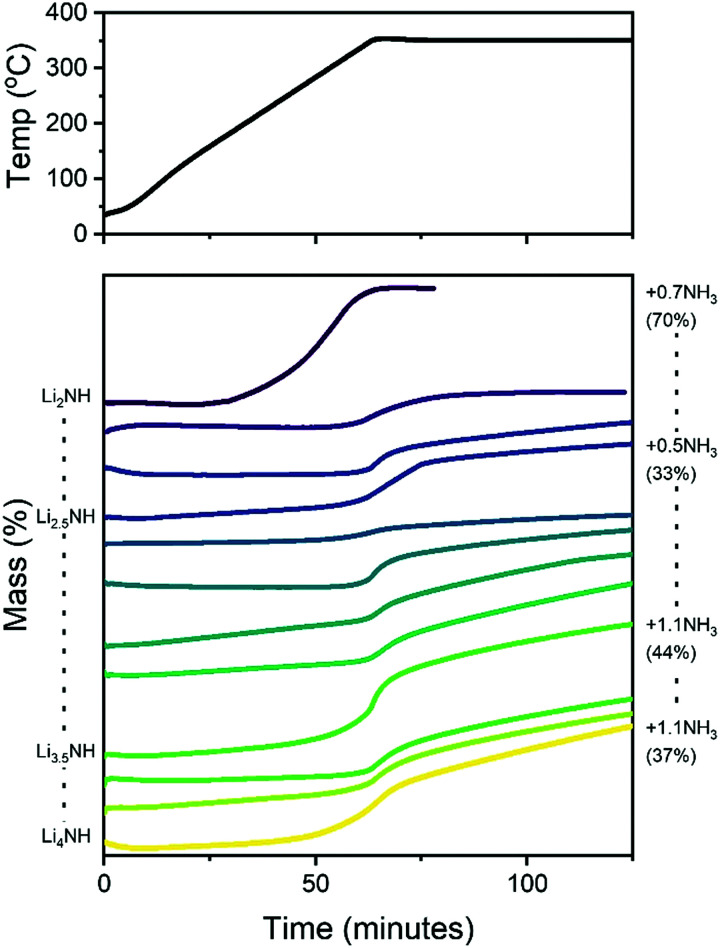
Simultaneous thermal analysis data for lithium imide–nitride–hydride samples heated under a constant flow of ammonia. Indicated on the right are the upper bounds of absorbed molar equivalents of ammonia by selected samples (assuming all mass gain is through ammonia absorption), along with the extent of reaction towards lithium amide.

It is therefore of significant interest the extent to which these compositional modifications perturb the electronic structure and reactivity of the materials. An initial demonstration of such variation is shown in Fig. 8, which details the results of STA experiments for the imide–nitride–hydride samples, heated under flowing ammonia. All of the samples would be expected to react to form lithium amide, by absorption of between one (for Li_2_NH) and three (for Li_4_NH) equivalents of ammonia. Indeed, mass uptake is observed. However, it is clear that even very minor incorporation of nitride–hydride into the solid solution leads to a dramatic change in the absorption profile, which more strongly resembles that of lithium nitride–hydride. The onset of rapid ammonia reaction was shifted by more than 100 °C and a generally slower kinetic profile can be seen, evidenced by the significantly reduced extent of reaction of the nitride–hydride containing samples over the duration of the experiment. Imide to amide conversion is likely to be facilitated by Grotthuss-style proton transfer through the solid, while conversion of nitride–hydride appears to be more kinetically hindered. These results are not inconsistent with the hypothesised stabilisation of nitride–hydride regions in lithium imide under ammonia decomposition conditions.^19^ A more comprehensive assessment of reactivity–composition relationship, including under catalytic conditions, is now possible with this library of compounds. As illustrated in Fig. 7, changing anion compositions will bring particular defect/vacancy/coordination features which could be targeted for particular applications.

## Conclusions

Through the synthesis of mixtures of lithium amide, lithium imide and lithium nitride–hydride, a solid solution with wide-ranging anion composition has been revealed. A highly disordered, anti-fluorite structure was observed for samples from majority lithium amide through to pure lithium imide and majority lithium nitride–hydride, with evidence for greater nitride–hydride incorporation at high temperatures. In contrast, the tetragonal lithium amide and nitride–hydride structures were found to show only limited evidence of compositional variation.

While imide–nitride–hydride solid solution formation is somewhat kinetically hindered, Raman spectroscopy of amide–imide samples was found to show monotonic variation in the local NH_*x*_ environment. Further work focused on other structural and spectroscopic probes of the local structure, along with computational investigations, will be able to provide further insight as to the relationship between local structural features in solid solutions and the parent phases.

The formation of solid solutions has been related to a number of key functional properties of the materials, from a composition range for maintaining the more highly-conductive cubic structure for optimising hydrogen storage characteristics, to broadening the thermal stability of the material, and demonstrating significant changes in the reactivity of lithium imide with ammonia upon small amounts of nitride–hydride incorporation. With the ability to select the desired anion mixture and its related vacancy and defect phenomena, the Li–N–H system now represents a rich library of candidates for materials applications in catalysis and energy storage, and a basis for expanded investigations into composition control in M–N–H materials.

## Author contributions

Conceptualisation: JWM, WIFD and TJW. Investigation: JMB, ASM, JWM, TJW and CAM. Formal analysis: JMB, ASM, JWM, TJW and WIFD. Software: TJW. Writing – original draft: JWM, JMB, ASM. Writing – review and editing: all authors.

## Conflicts of interest

There are no conflicts of interest to declare.

## Supplementary Material

CP-023-D1CP02440J-s001
