# Supplementary material for: Compositional flexibility in Li–N–H materials: implications for ammonia catalysis and hydrogen storage
Source: Phys Chem Chem Phys. 2021 Jul 5;23(28):15091–100. doi: 10.1039/d1cp02440j (PMC8294645; doi:10.1039/d1cp02440j)
Supplement: CP-023-D1CP02440J-s001 [file CP-023-D1CP02440J-s001.pdf]

Electronic Supplementary Material (ESI) for Physical Chemistry Chemical Physics.
This journal is © the Owner Societies 2021

Supplementary information for:

Compositional flexibility in Li-N-H materials: implications for ammonia catalysis and hydrogen storage.

1 – Experimental data for $LiNH_2$ + LiH reactions 2

2 – Fitted XRD patterns 6

3 – Rietveld model for imide-nitride-hydride solid solution 15

4 – Rietveld model for $P\overline{4}$ $LiNH_2$ structure 17

5 – Raman spectra fitting results 18

1 - Experimental data for $LiNH_2$ + LiH reactions

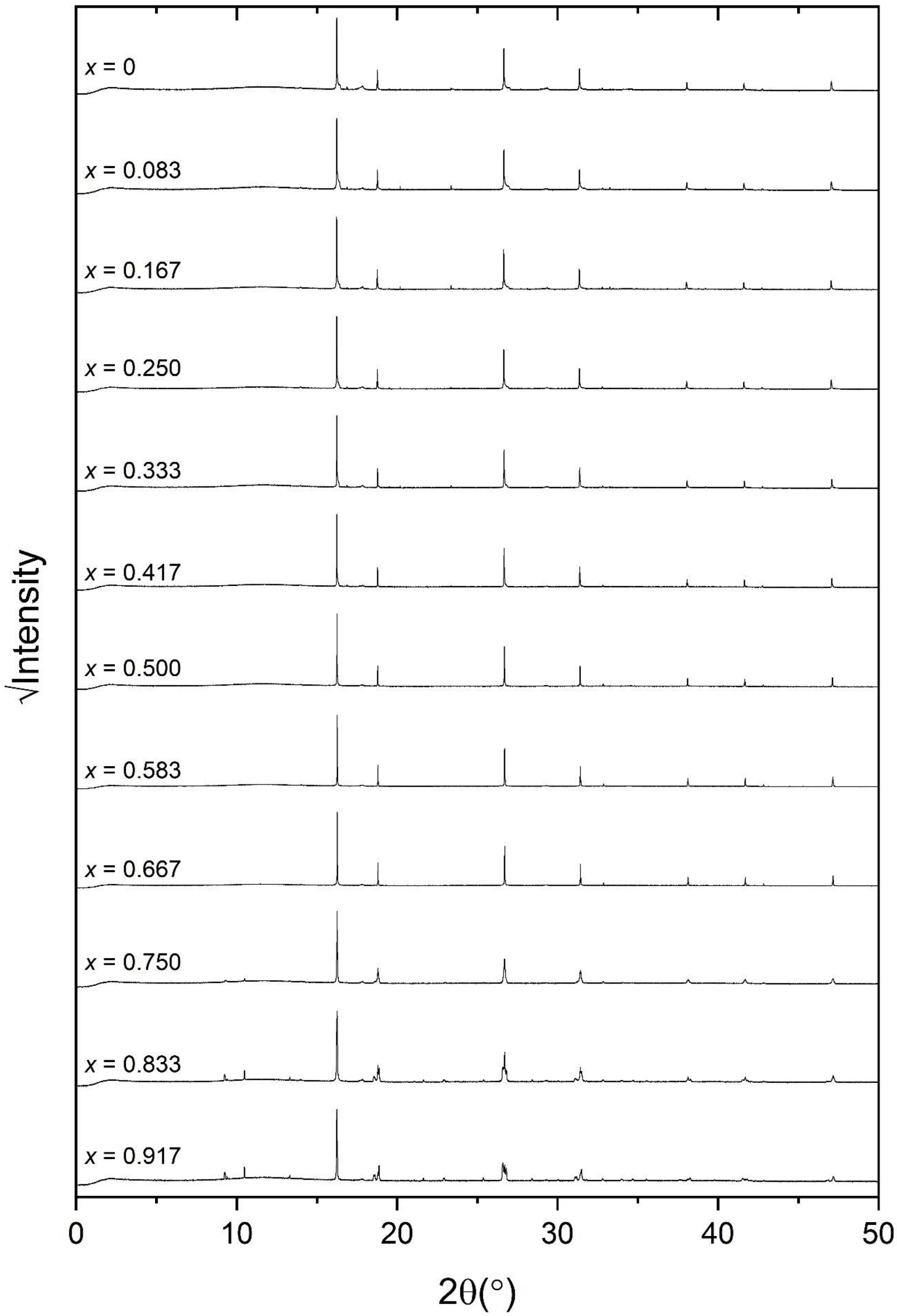

Figure S1 – Synchrotron X-ray diffraction data for $Li_{2-x}NH_{1+x}$ samples produced by the reaction of lithium amide and lithium hydride.

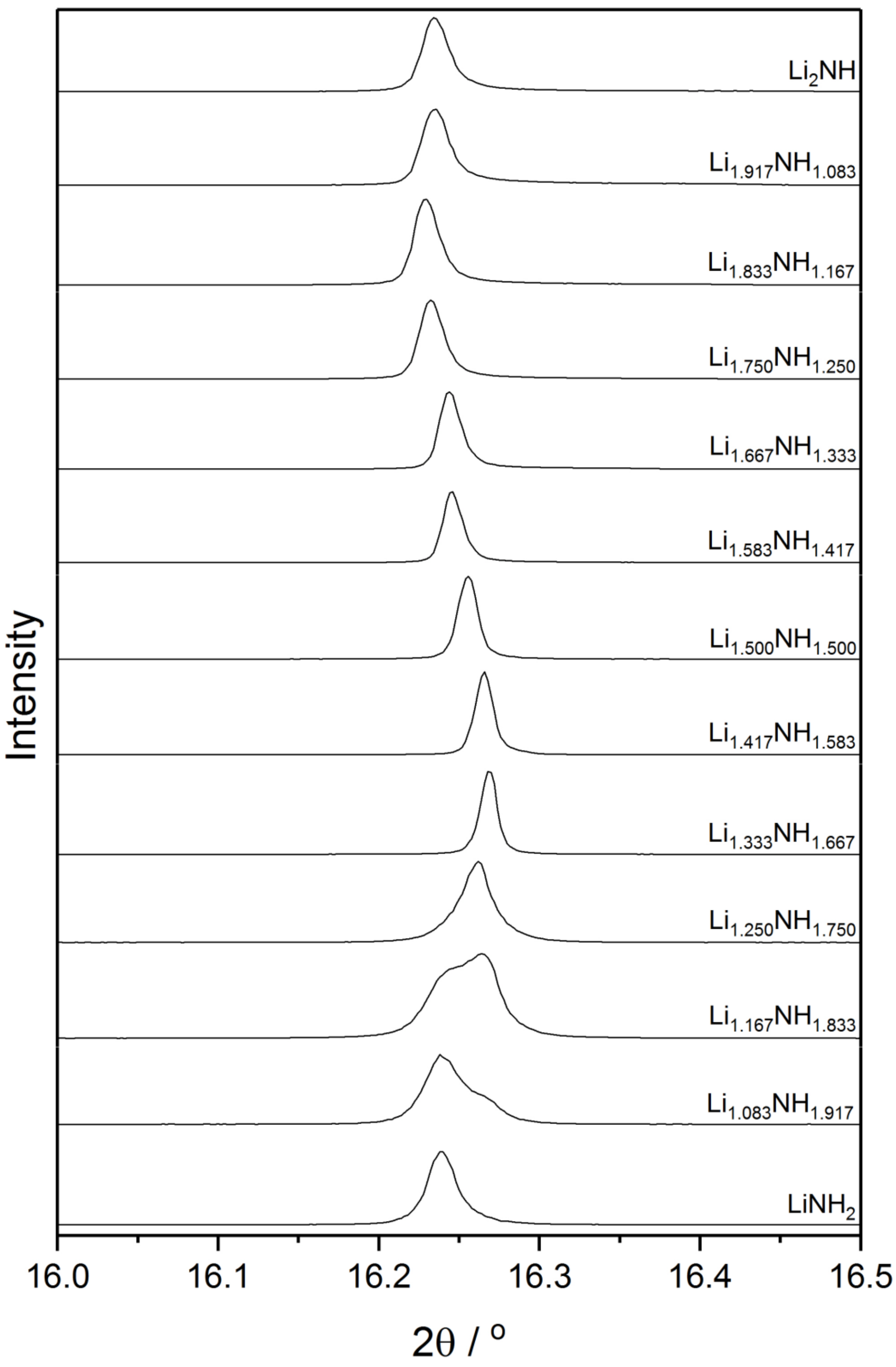

Figure S2 – Segment of synchrotron X-ray diffraction data for $Li_{2-x}NH_{1+x}$ samples produced by the reaction of lithium amide and lithium hydride.

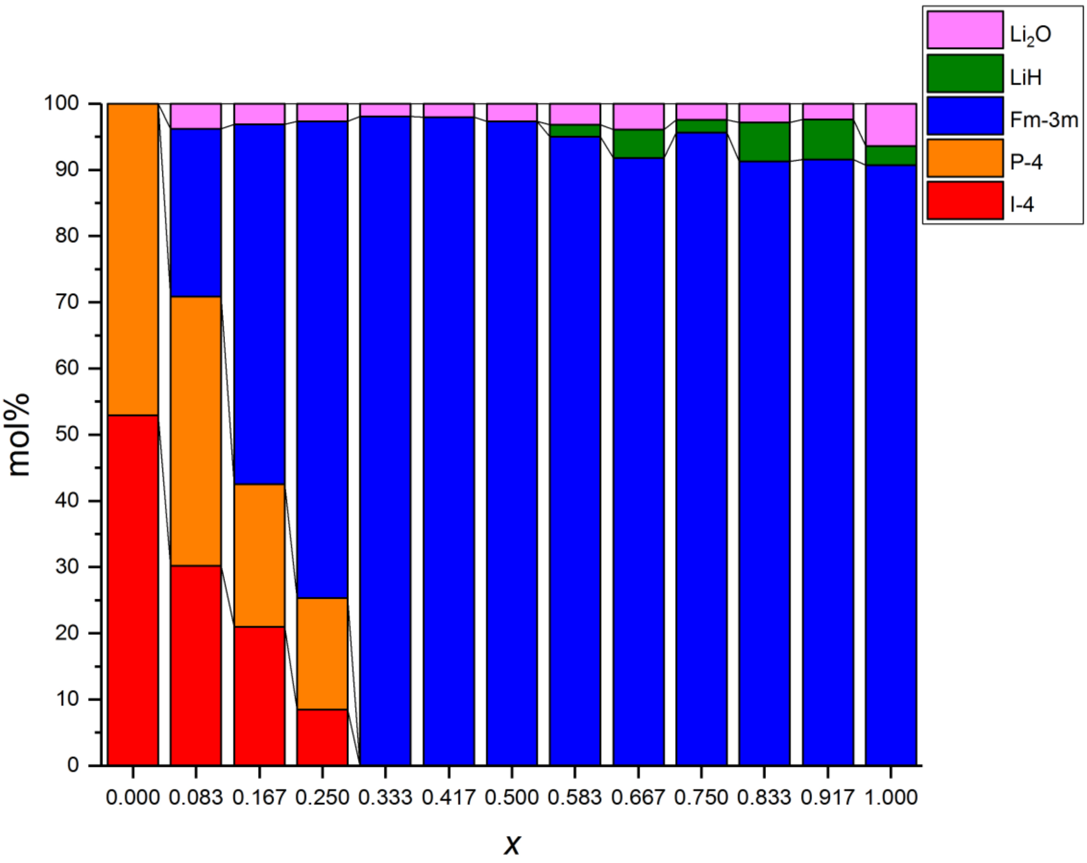

Figure S3 – Refined molar composition of $Li_{2-x}NH_{1+x}$ samples produced by the reaction of lithium amide and lithium hydride as assessed by Rietveld analysis of synchrotron X-ray diffraction data.

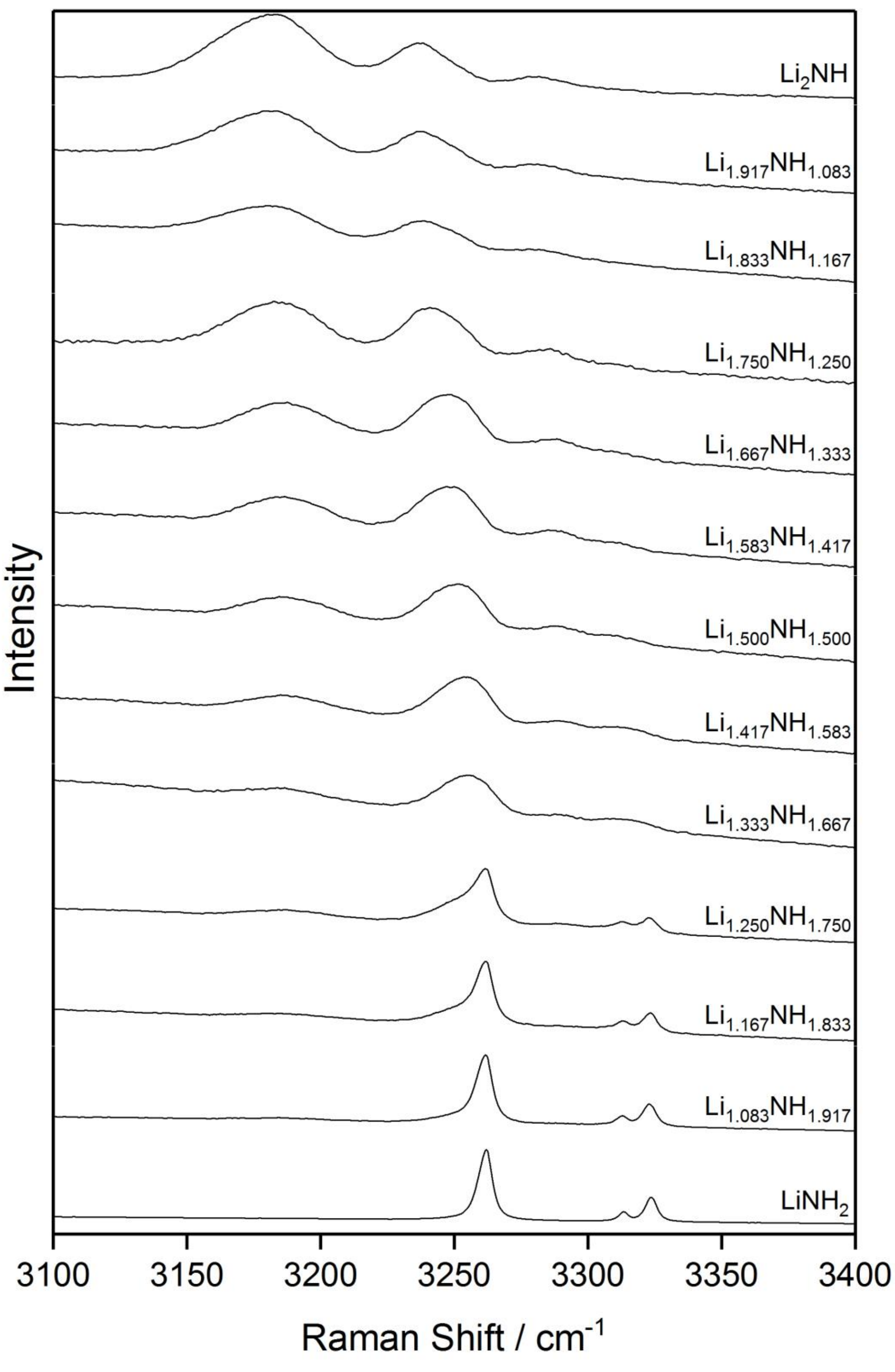

Figure S4 – Raman data for $Li_{2-x}NH_{1+x}$ samples produced by the reaction of lithium amide and lithium hydride.

2 - Fitted XRD patterns

Tick mark colours:

$P\bar{4}$ $LiNH_2$ = dark green $I\bar{4}$ $LiNH_2$ = light green $Fm\bar{3}m$ solid solution / $Fd\bar{3}m$ Li_2NH = orange

Li_2O = magenta

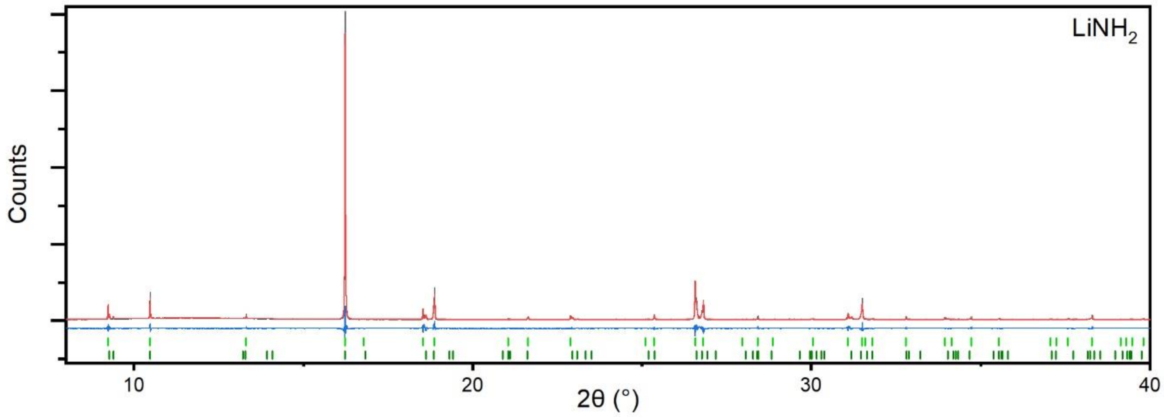

Figure S5 - Synchrotron X-ray diffraction data for $LiNH_2$ sample with data shown in black, fit by Rietveld analysis in red, and difference in blue.

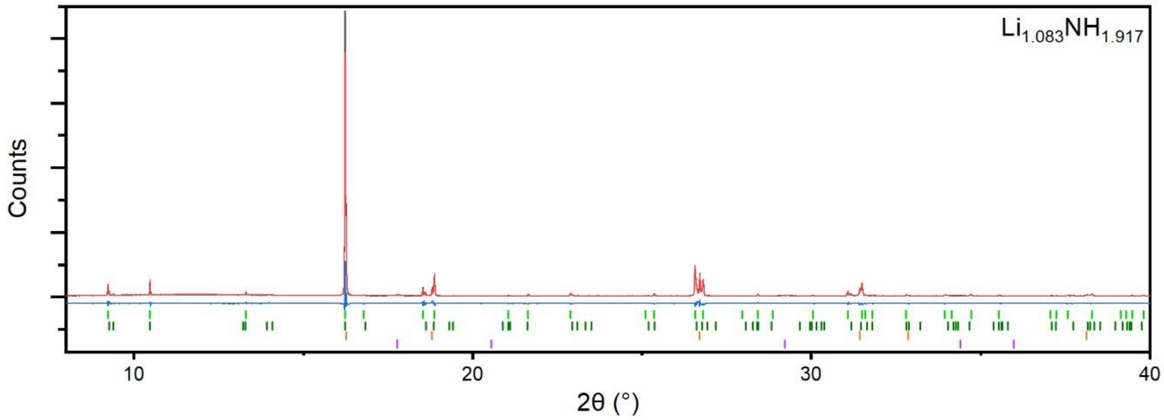

Figure S6 - Synchrotron X-ray diffraction data for $Li_{1.083}NH_{1.917}$ sample with data shown in black, fit by Rietveld analysis in red, and difference in blue.

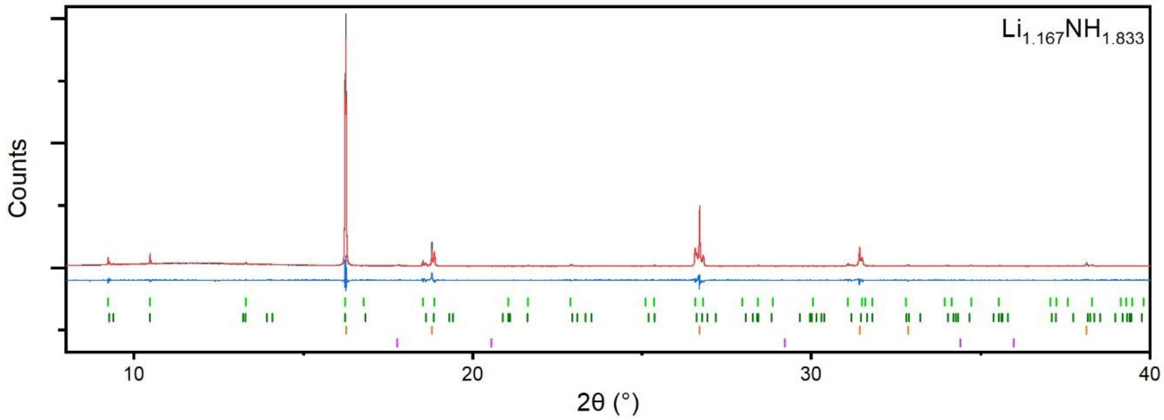

Figure S7 - Synchrotron X-ray diffraction data for $Li_{1.167}NH_{1.833}$ sample with data shown in black, fit by Rietveld analysis in red, and difference in blue.

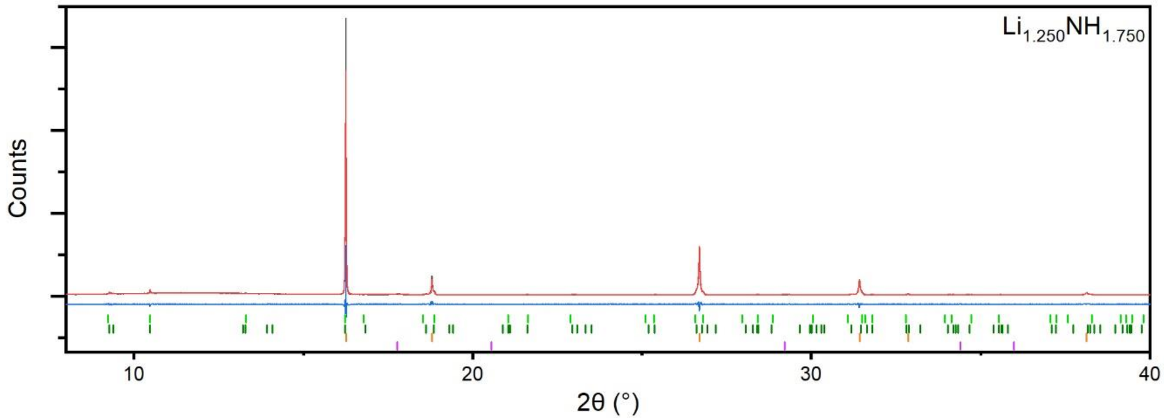

Figure S8 - Synchrotron X-ray diffraction data for $Li_{1.250}NH_{1.750}$ sample with data shown in black, fit by Rietveld analysis in red, and difference in blue.

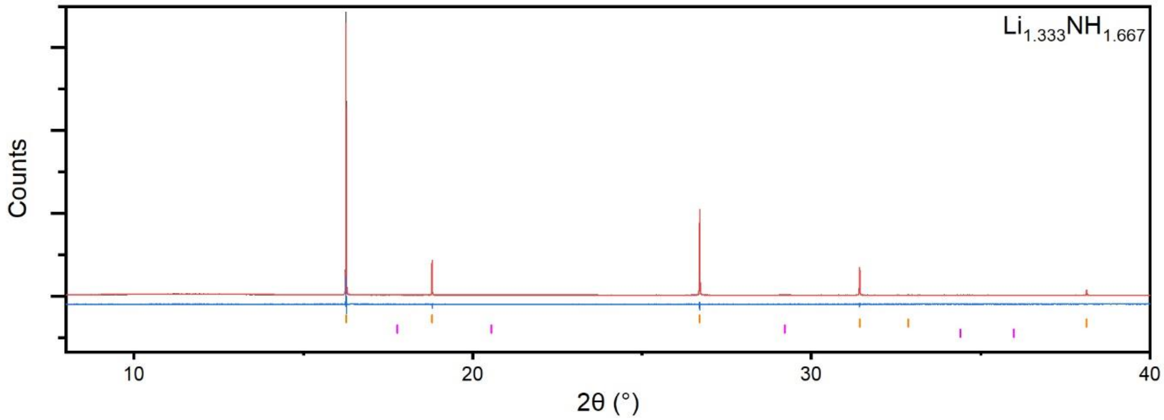

Figure S9 - Synchrotron X-ray diffraction data for $Li_{1.333}NH_{1.667}$ sample with data shown in black, fit by Rietveld analysis in red, and difference in blue.

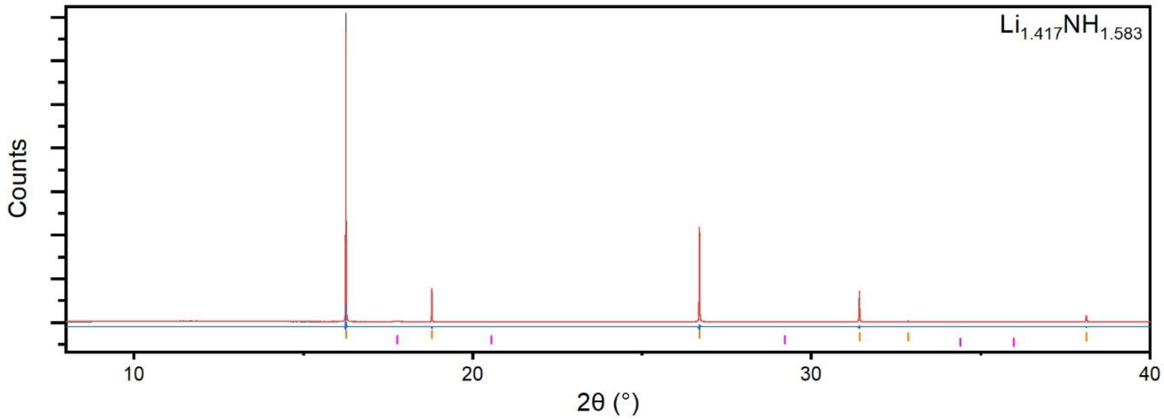

Figure S10 - Synchrotron X-ray diffraction data for $Li_{1.417}NH_{1.583}$ sample with data shown in black, fit by Rietveld analysis in red, and difference in blue.

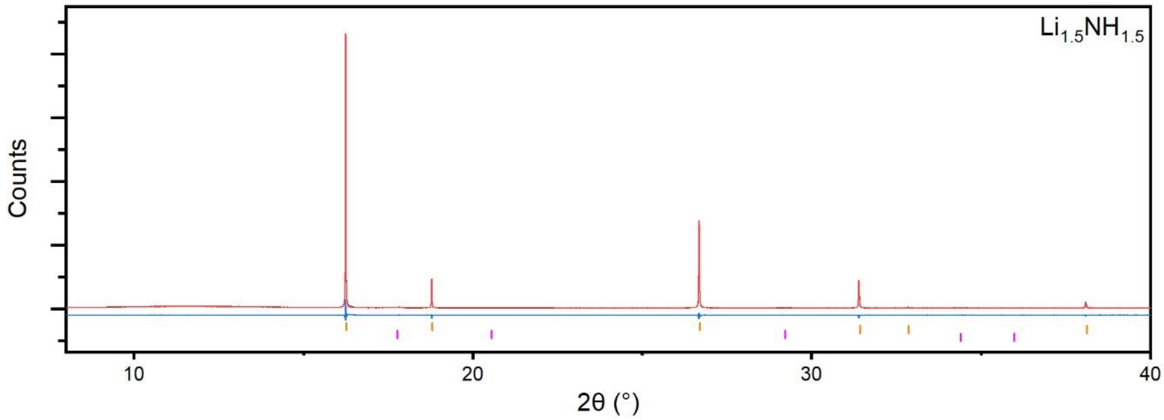

Figure S11 - Synchrotron X-ray diffraction data for $Li_{1.5}NH_{1.5}$ sample with data shown in black, fit by Rietveld analysis in red, and difference in blue.

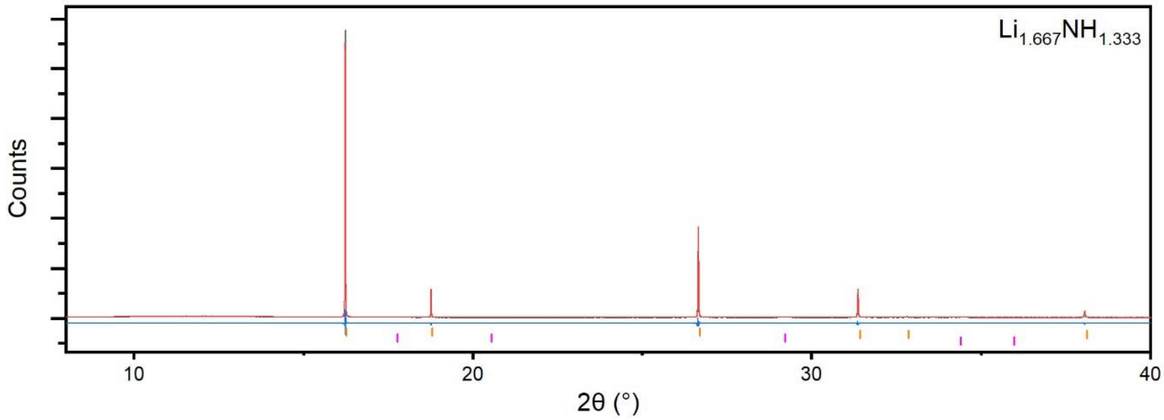

Figure S12 - Synchrotron X-ray diffraction data for $Li_{1.667}NH_{1.333}$ sample with data shown in black, fit by Rietveld analysis in red, and difference in blue.

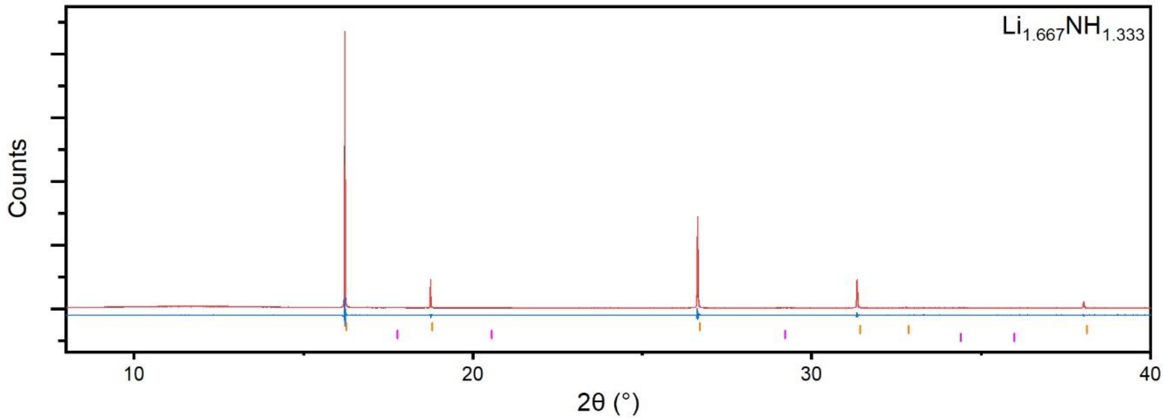

Figure S13 - Synchrotron X-ray diffraction data for $Li_{1.667}NH_{1.333}$ sample with data shown in black, fit by Rietveld analysis in red, and difference in blue.

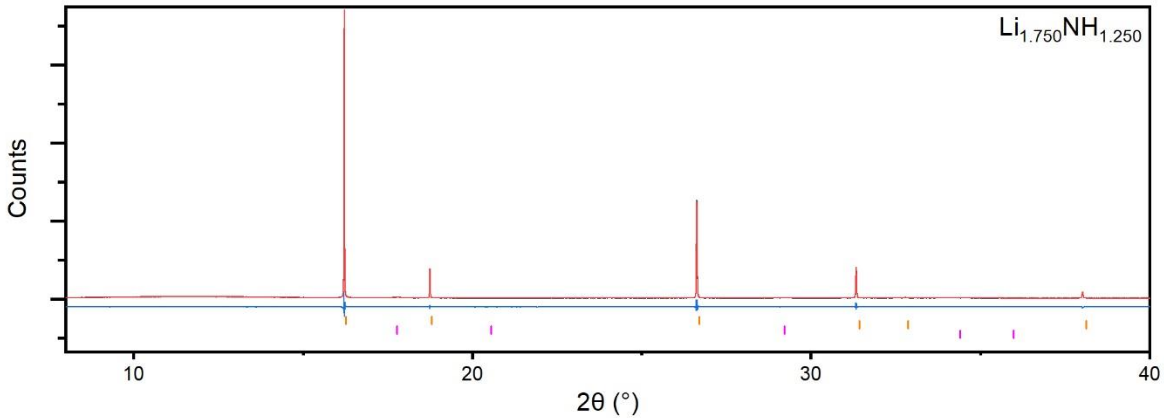

Figure S14 - Synchrotron X-ray diffraction data for $Li_{1.750}NH_{1.250}$ sample with data shown in black, fit by Rietveld analysis in red, and difference in blue.

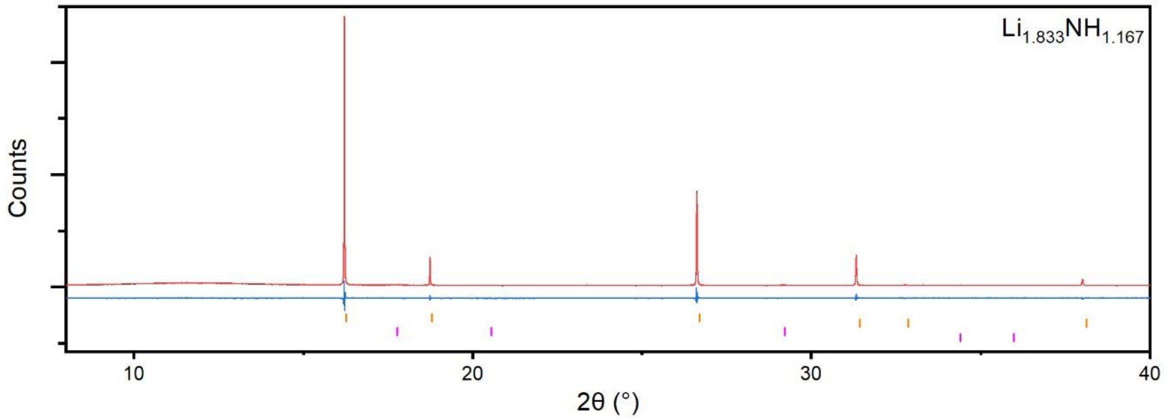

Figure S15 - Synchrotron X-ray diffraction data for $Li_{1.833}NH_{1.167}$ sample with data shown in black, fit by Rietveld analysis in red, and difference in blue.

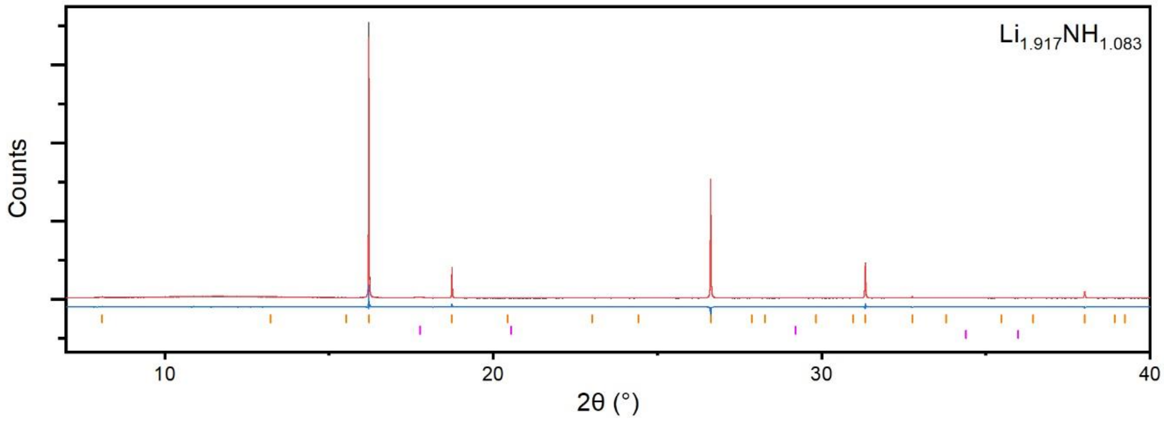

Figure S16 - Synchrotron X-ray diffraction data for $Li_{1.917}NH_{1.083}$ sample with data shown in black, fit by Rietveld analysis in red, and difference in blue.

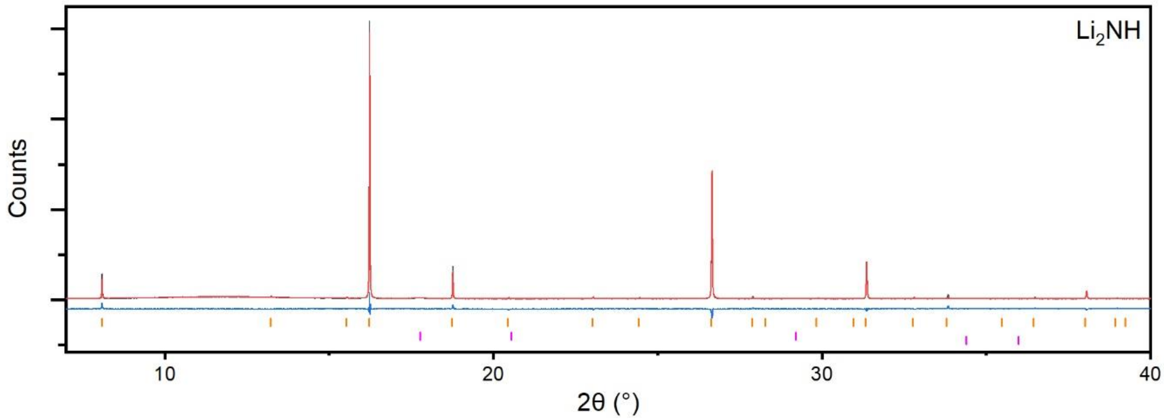

Figure S17 - Synchrotron X-ray diffraction data for Li_2NH sample with data shown in black, fit by Rietveld analysis in red, and difference in blue.

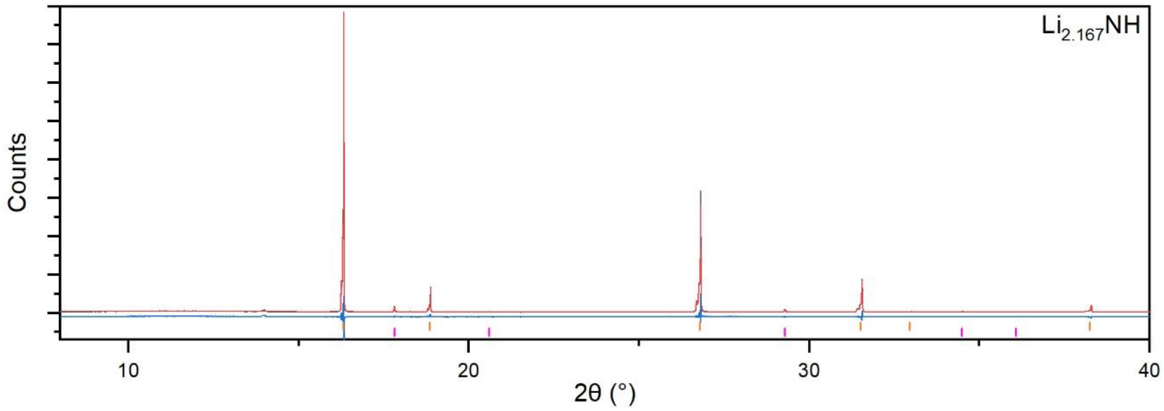

Figure S18 - Synchrotron X-ray diffraction data for $Li_{2.167}NH$ sample with data shown in black, fit by Rietveld analysis in red, and difference in blue.

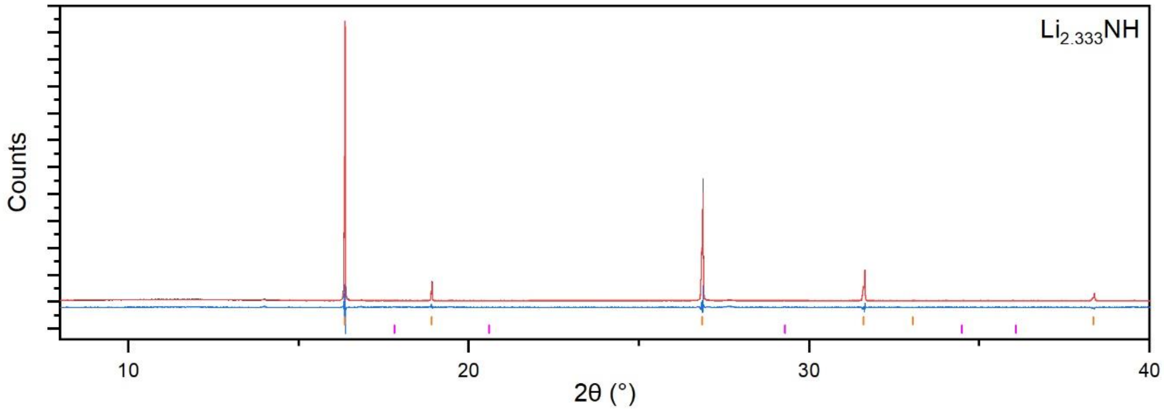

Figure S19 - Synchrotron X-ray diffraction data for $Li_{2.333}NH$ sample with data shown in black, fit by Rietveld analysis in red, and difference in blue.

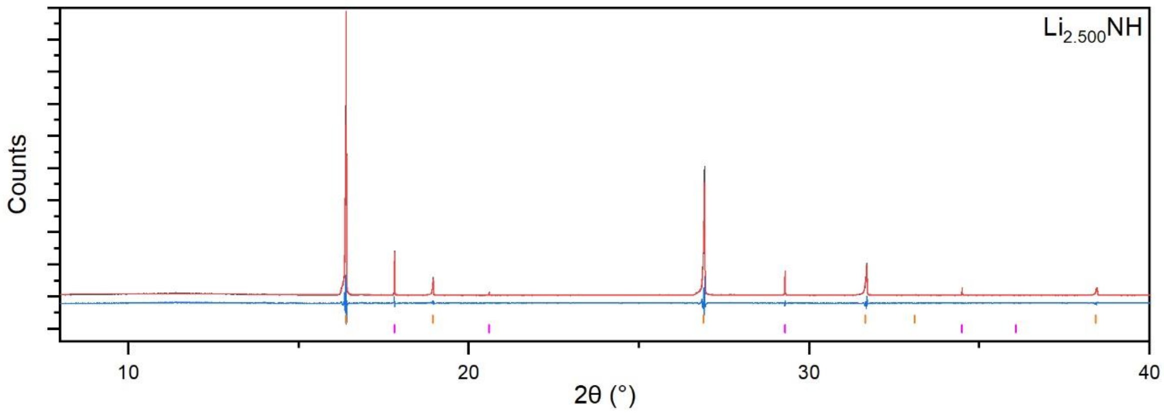

Figure S20 - Synchrotron X-ray diffraction data for $Li_{2.5}NH$ sample with data shown in black, fit by Rietveld analysis in red, and difference in blue.

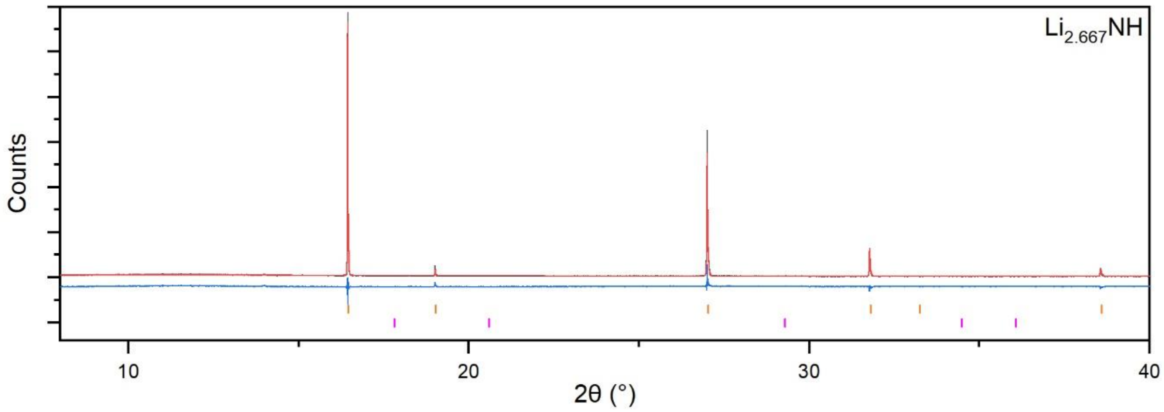

Figure S21 - Synchrotron X-ray diffraction data for $Li_{2.667}NH$ sample with data shown in black, fit by Rietveld analysis in red, and difference in blue.

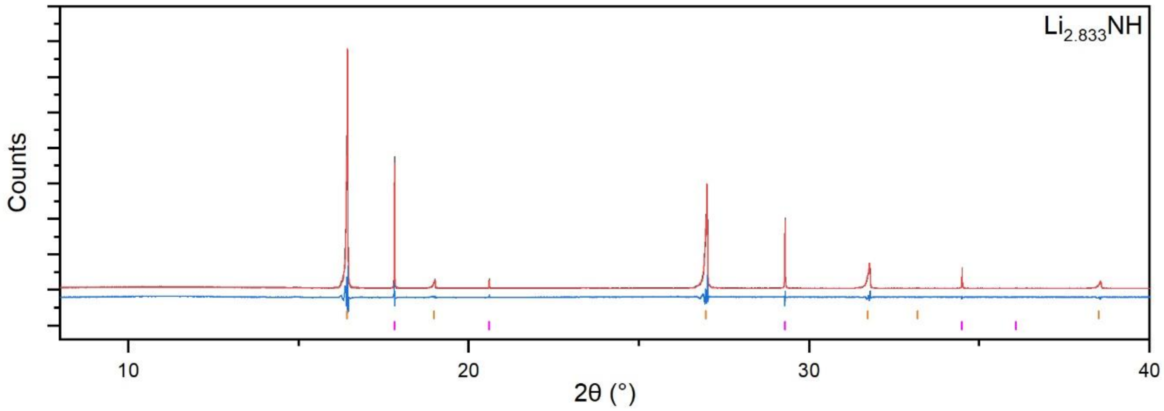

Figure S22 - Synchrotron X-ray diffraction data for $Li_{2.833}NH$ sample with data shown in black, fit by Rietveld analysis in red, and difference in blue.

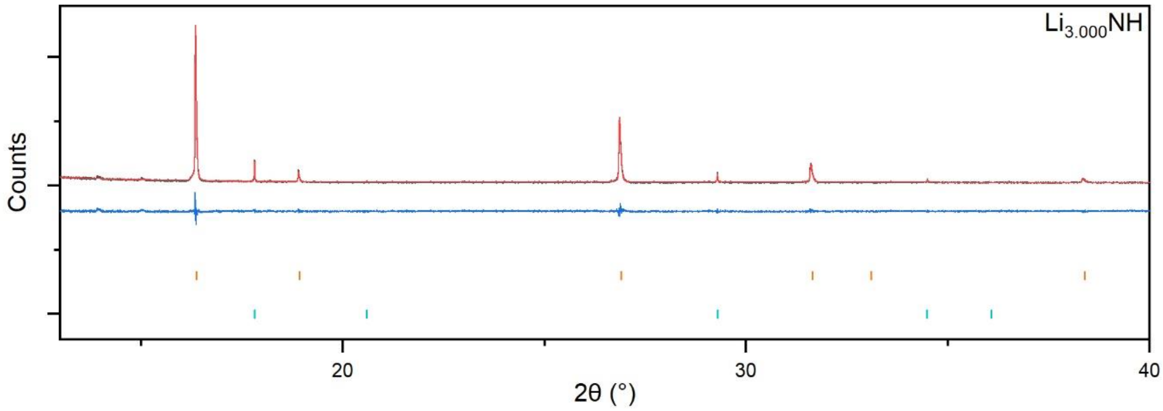

Figure S23 - Synchrotron X-ray diffraction data for Li$_3$NH sample with data shown in black, fit by Rietveld analysis in red, and difference in blue.

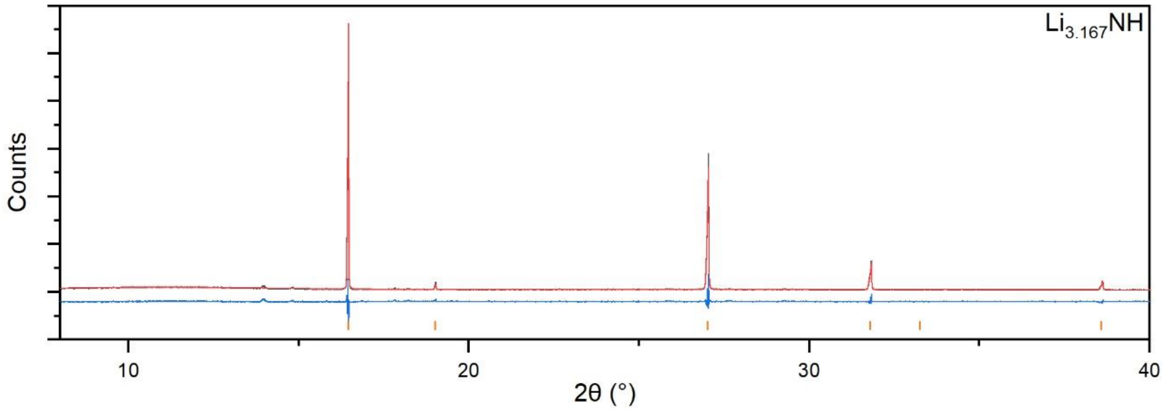

Figure S24 - Synchrotron X-ray diffraction data for Li$_{3.167}$NH sample with data shown in black, fit by Rietveld analysis in red, and difference in blue.

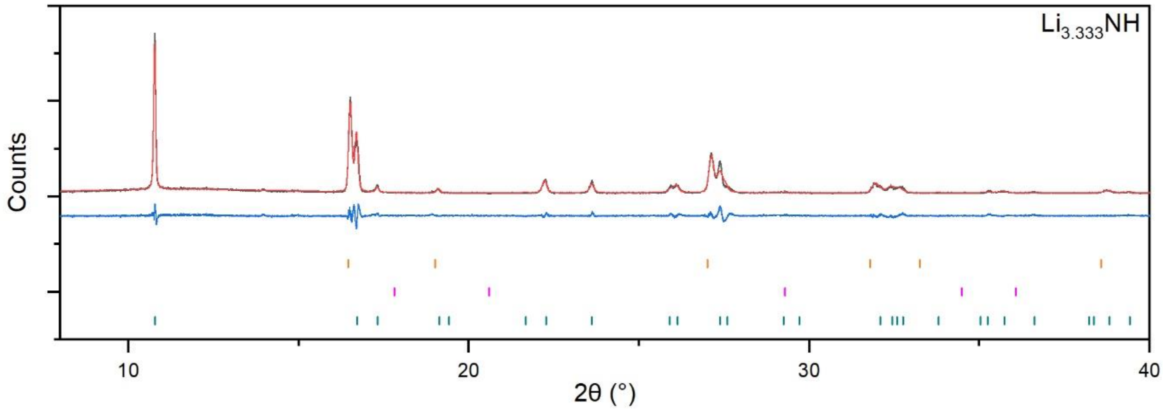

Figure S25 - Synchrotron X-ray diffraction data for Li$_{3.333}$NH sample with data shown in black, fit by Rietveld analysis in red, and difference in blue.

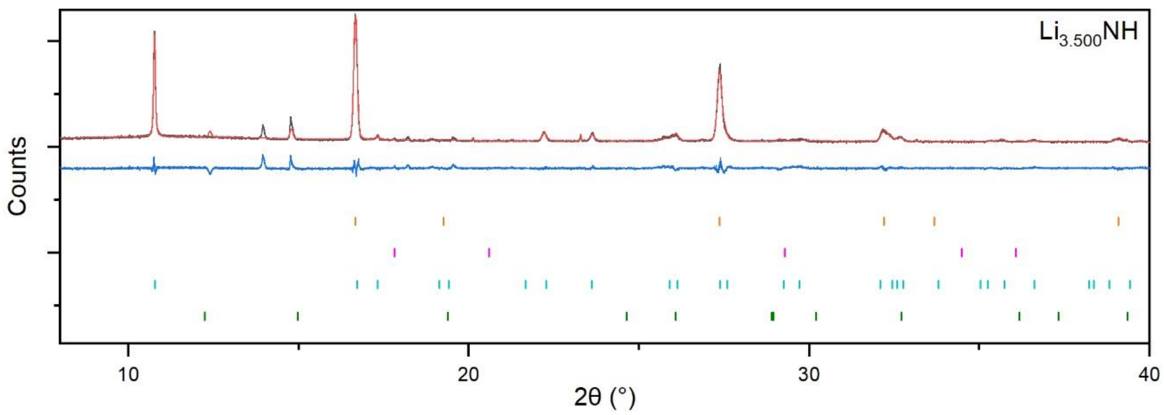

Figure S26 - Synchrotron X-ray diffraction data for $Li_{3.500}NH$ sample with data shown in black, fit by Rietveld analysis in red, and difference in blue.

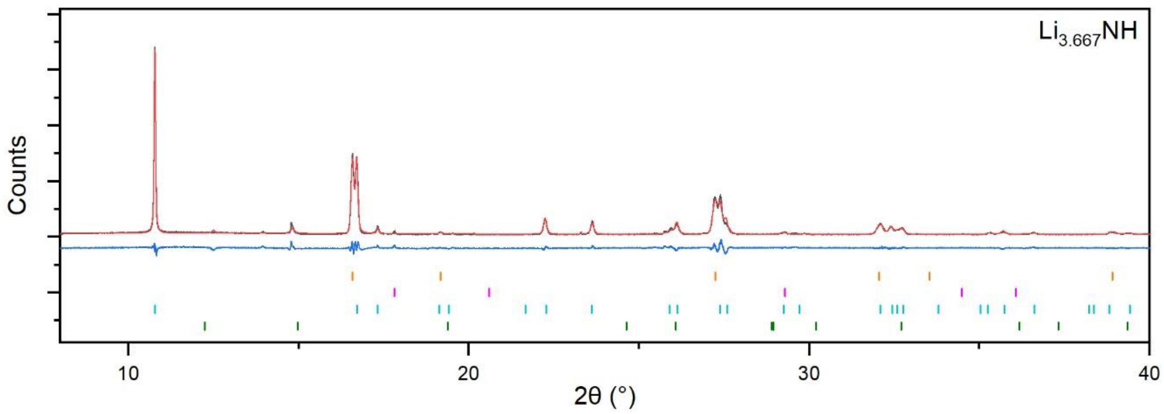

Figure S27 - Synchrotron X-ray diffraction data for $Li_{3.667}NH$ sample with data shown in black, fit by Rietveld analysis in red, and difference in blue.

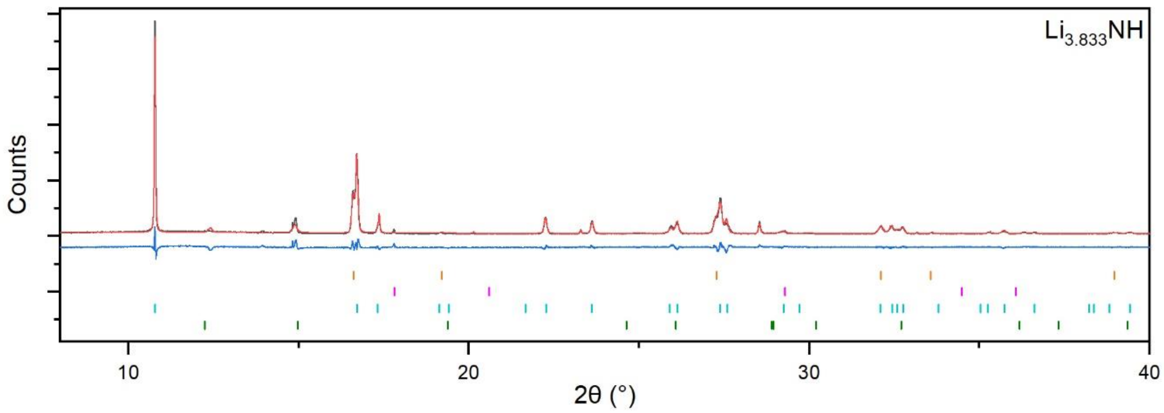

Figure S28 - Synchrotron X-ray diffraction data for $Li_{3.833}NH$ sample with data shown in black, fit by Rietveld analysis in red, and difference in blue.

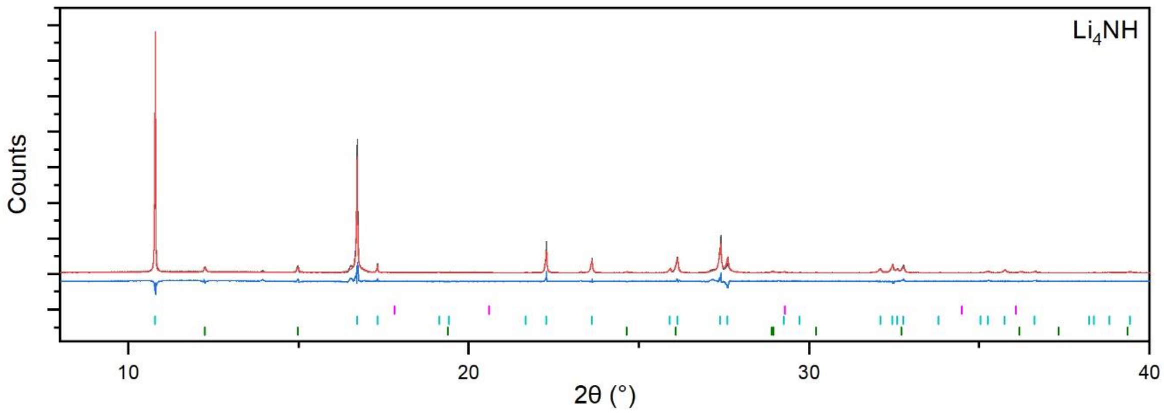

Figure S29 - Synchrotron X-ray diffraction data for Li_4NH sample with data shown in black, fit by Rietveld analysis in red, and difference in blue.

3 – Rietveld model for imide-nitride-hydride solid solution

A modified $Fm\bar{3}m$ lithium imide CIF was created to model the imide-nitride-hydride solid solution structure, in which additional atomic positions were added to account for the N^{3-} and H^{-} ions at (0, 0, 0) from lithium nitride hydride incorporation. The following restraints to the site occupancy were incorporated. Since the total occupancy, Occtot, of this site must be equal to one, the following assumption can be made:

$Occ_{tot} = 1 = Occ_{N(NH)} + Occ_{N3-} + Occ_{H-}$

where

$Occ_{N3-} = Occ_{H-}$

and therefore,

$Occ_{N(NH)} = 1 - 2Occ_{H-}$

This file was then used to perform a Rietveld analysis on the sample while keeping the thermal parameters for the three anions equal.

For the more complex peak shapes for imide-rich solid solutions, the solid solution was modelled as a series of these phases where the maximum and minimum values for *a* and Occ_{NH} were set using the values obtained from the unconstrained refinement of the first and last solid solution samples: $Li_{2.17}NH$ and $Li_{3.17}NH$:

$a_{max} = 5.051$, $a_{min} = 4.976$ and $Occ_{Nmax} = 1$, $Occ_{Nmin} = 0.135$

Intermediate phases were then included assuming a Vegard's law type behaviour between the lattice parameter and composition:

$a_n = a_{max} - x_n(a_{max} - a_{min})$ (4.6)
$Occ_{Nn} = Occ_{Nmax} – x_n(Occ_{Nmax} - Occ_{Nmin})$

Consistent size, strain and thermal ellipsoids were used across the multi-phase model. An example of the fit using this model is shown in Figure S30.

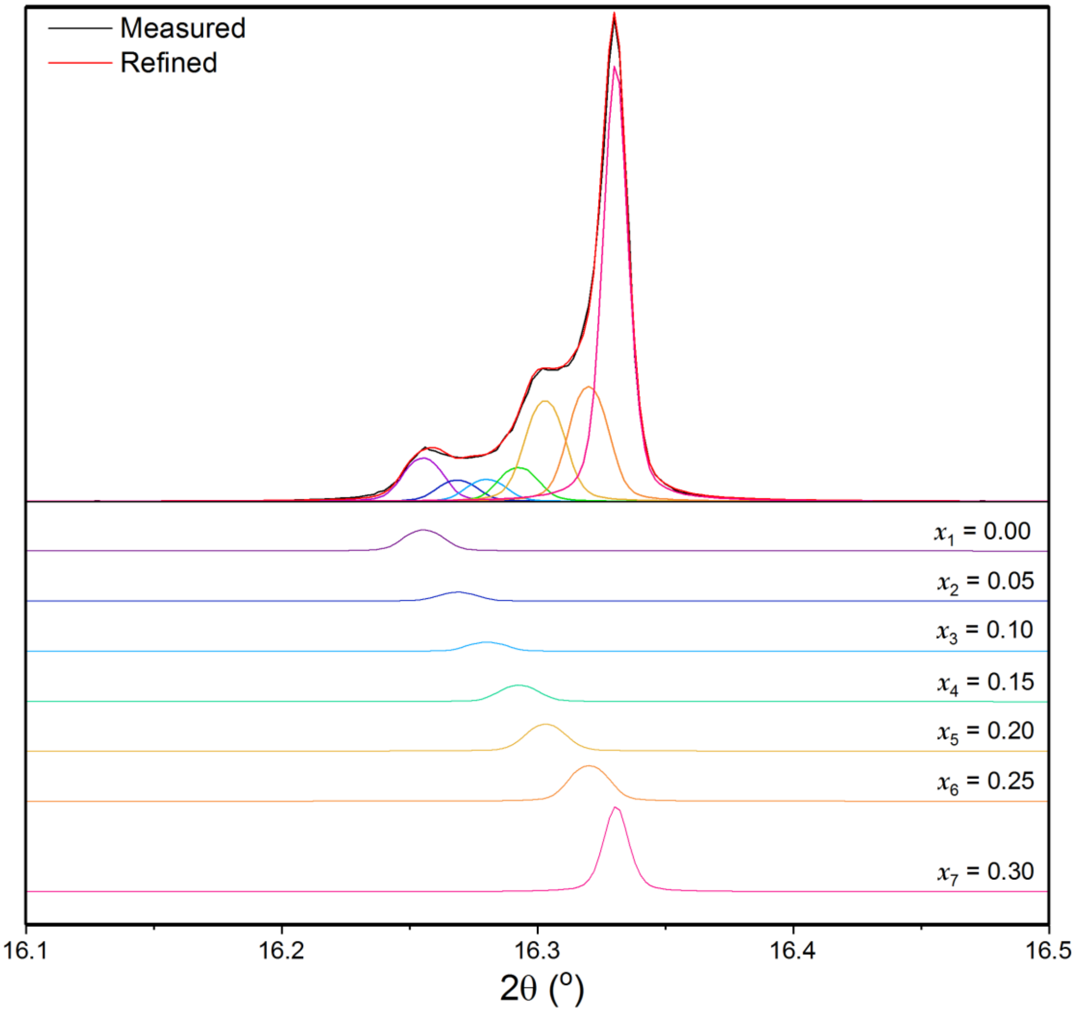

Figure S30 – An example of the fit to the (111) of the $Li_{2.167}NH$ sample using the multi-phase model.

4 – Rietveld model for $P\overline{4}$ $LiNH_2$ structure

Table S1 – A summary of Rietveld analysis of $P\overline{4}$ lithium amide.

a / Å	5.04563(4)	
c / Å	10.2175(1)	
Site	(*x*, *y*, *z*)	Occupancy
Li1	(0, 0, 0)	1.0(2)
Li2	(0.5, 0.5, 0.5)	0.5(2)
Li3	(0, 0.5, 0.75)	1.0(2)
Li4	(0, 0.5, 0)	0.9(2)
Li5	(0, 0.5, 0.5)	0.8(2)
Li6	(0, 0, 0.25)	0.3(2)
N1	(-0.226, -0.240, -0.111)	1
N2	(-0.729, -0.759, -0.614)	1
H1	(-0.222, -0.174, -0.192)	1
H2	(-0.722, -0.674, -0.692)	1
H3	(-0.365, -0.363, -0.119)	1
H4	(-0.865, -0.863, -0.619)	1

5 – Raman spectra fitting results

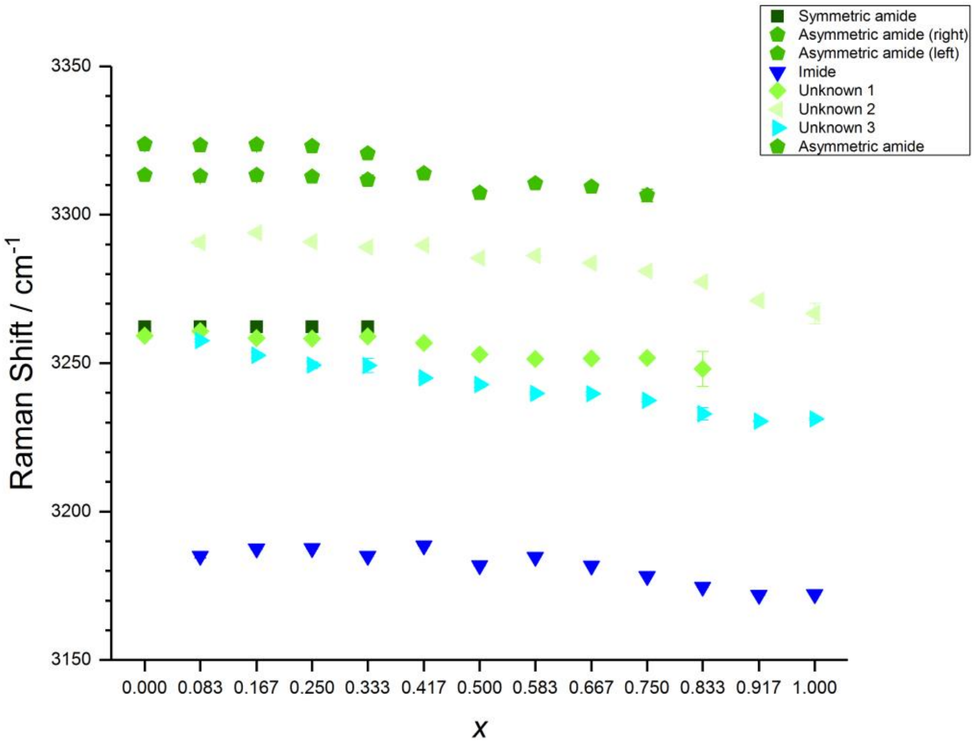

Figure S31 - Trends in Raman peak positions for $Li_{1+x}NH_{2-x}$ prepared by the nitride reaction.

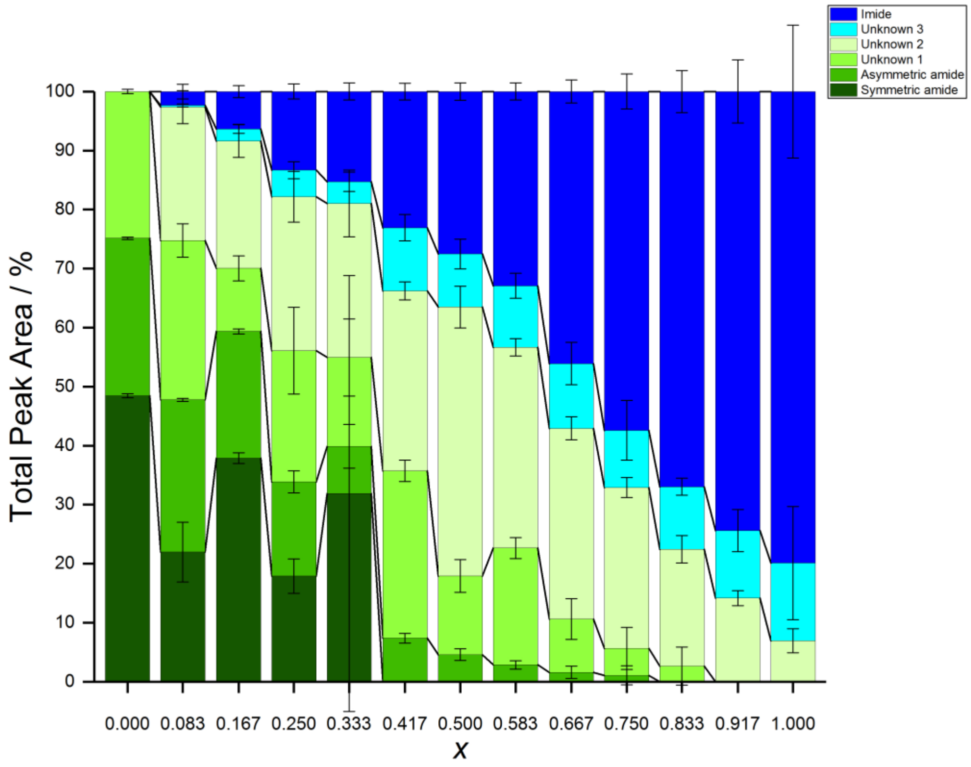

Figure S32 - Column stack plots representing the quantity of each Raman peak area as a percentage of the total peak area for each value of x, where $Li_{1+x}NH_{2-x}$ samples prepared by the nitride reaction.
